# ASM-3 Acid Sphingomyelinase Functions as a Positive Regulator of the DAF-2/AGE-1 Signaling Pathway and Serves as a Novel Anti-Aging Target

**DOI:** 10.1371/journal.pone.0045890

**Published:** 2012-09-25

**Authors:** Yongsoon Kim, Hong Sun

**Affiliations:** 1 Laboratory of Cancer Genomics, Nevada Cancer Institute, Las Vegas, Nevada, United States of America; 2 Department of Chemistry, University of Nevada, Las Vegas, Las Vegas, Nevada, United States of America; VIB & Katholieke Universiteit Leuven, Belgium

## Abstract

In *C. elegans*, the highly conserved DAF-2/insulin/insulin-like growth factor 1 receptor signaling (IIS) pathway regulates longevity, metabolism, reproduction and development. In mammals, acid sphingomyelinase (ASM) is an enzyme that hydrolyzes sphingomyelin to produce ceramide. ASM has been implicated in CD95 death receptor signaling under certain stress conditions. However, the involvement of ASM in growth factor receptor signaling under physiological conditions is not known. Here, we report that *in vivo* ASM functions as a positive regulator of the DAF-2/IIS pathway in *C. elegans*. We have shown that inactivation of *asm-3* extends animal lifespan and promotes dauer arrest, an alternative developmental process. A significant cooperative effect on lifespan is observed between *asm-3* deficiency and loss-of-function alleles of the *age-1*/PI 3-kinase, with the *asm-3; age-1* double mutant animals having a mean lifespan 259% greater than that of the wild-type animals. The lifespan extension phenotypes caused by the loss of *asm-3* are dependent on the functions of *daf-16*/FOXO and *daf-18*/PTEN. We have demonstrated that inactivation of *asm-3* causes nuclear translocation of DAF-16::GFP protein, up-regulates endogenous DAF-16 protein levels and activates the downstream targeting genes of DAF-16. Together, our findings reveal a novel role of *asm-3* in regulation of lifespan and diapause by modulating IIS pathway. Importantly, we have found that two drugs known to inhibit mammalian ASM activities, desipramine and clomipramine, markedly extend the lifespan of wild-type animals, in a manner similar to that achieved by genetic inactivation of the *asm* genes. Our studies illustrate a novel strategy of anti-aging by targeting ASM, which may potentially be extended to mammals.

## Introduction

The nematode *Caenorhabditis elegans* serves as an excellent genetic model to study the mechanisms of cell signaling and organismal aging. In *C. elegans*, the DAF-2/IIS pathway plays a critical role in the regulation of animal lifespan, metabolism, reproduction and development [1]–[4]. In particular, reduced DAF-2/IIS signaling results in lifespan extension and stress resistance in adult animals, and dauer arrest in larvae [1]–[4]. The components of the IIS pathway are also highly conserved in various organisms including worms, flies and mammals [5]. In the IIS pathway, ligand engagement to the DAF-2 receptor tyrosine kinase, through activating the AGE-1/PI-3 kinase homolog, leads to the activation of PDK-1 and then AKT-1/2 serine/threonine kinases [3], [4]. Activated AKT-1/2 phosphorylates the DAF-16/FOXO transcription factor, preventing the latter from entering into the nucleus [3], [6]–[8]. Down-regulation of DAF-2 signaling leads to nuclear translocation and activation of DAF-16 [3], [6], [9]. Loss of *daf-16* suppresses the phenotypes of lifespan extension and constitutive dauer arrest in the mutants defective in *daf-2* signaling [1]–[3], [10]. On the other hand, increased protein levels of DAF-16, either through transgene-mediated overexpression or through reduced protein degradation, extends animal lifespan [11]–[13]. Another negative regulator of the *daf-2* pathway, *daf-18*, encoding the *C. elegans* homolog of human PTEN tumor suppressor, antagonizes *daf-2* and *age-1* signaling. Thus, inactivation of *daf-18* suppresses lifespan extension and constitutive dauer arrest phenotypes of *daf-2* or *age-1* mutants [14], [15]. However, additional regulators of the IIS pathway remain to be identified.

In mammals, acid sphingomyelinase (ASM) is a phosphodiesterase that hydrolyzes sphingomyelin to produce ceramide and phosphorylcholine [16]. When acting in lipid rafts, which are plasma membrane microdomains [17], ASM leads to the production of ceramide-enriched lipid rafts [18], [19]. Because ceramides have a physical property of self-association, it is likely that ceramide-enriched lipid rafts can provide a unique local microenvironment for protein-protein interactions. Indeed, ASM-dependent and ceramide-enriched lipid rafts have been shown to facilitate the oligomerization and signaling of CD95 receptors, leading to apoptosis [18], [19]. However, most recent studies have shown that CD95, previously known as death receptors, also possess pro-proliferation and pro-survival function *in vivo* under physiological conditions [20]. It is not known if ASM is involved in the pro-proliferation and pro-survival signaling of the CD95 receptors. In addition to ASM, neutral sphingomyelinase (NSM) can also hydrolyze sphingomyelin to produce ceramide, although the exact site of NSM action is not clear [21], [22]. Ceramides can also be synthesized by a *de novo* biosynthetic pathway, which occurs in the endoplastic reticulum and Golgi apparatus [21], [22]. Ceramides produced through biosynthesis are also known to affect the cellular processes such as cell signaling, stress resistance, and apoptosis, although the detailed molecular mechanisms are not clear [21], [22]. As ceramides produced by hydrolysis of sphingomyelin and through biosynthesis are localized in different cellular compartments, it is possible that they may have different cellular functions. In *C. elegans*, while the ceramides produced through *de novo* biosynthesis are known to be involved in the radiation-induced apoptosis in the germline [23], the roles of ceramides produced through hydrolysis of sphingomyelin are not clear, although *C. elegans* have been shown to contain ASM homologs but no NSM homologs [23], [24].

In the current study, we used a genetic approach to study the role of *asm* in signal transduction in the *C. elegans* model system. Our results show that *C. elegans* ASM homologs, in particular, ASM-3, is a critical and positive regulator of the DAF-2/IIS pathway and controls both animal lifespan and dauer formation. Using pharmacological agents, we further demonstrated that inhibition of CeASM, similar to genetic inactivation of the *asm* genes, leads to significant animal lifespan extension. Our studies illustrate a novel anti-aging strategy of targeting CeASMs to down-regulate the DAF-2/IIS pathway, which could potentially be extended to mammals.

## Results

### *asm* Genes Regulate Animal Lifespan as Novel Longevity Genes

**Figure 1:**
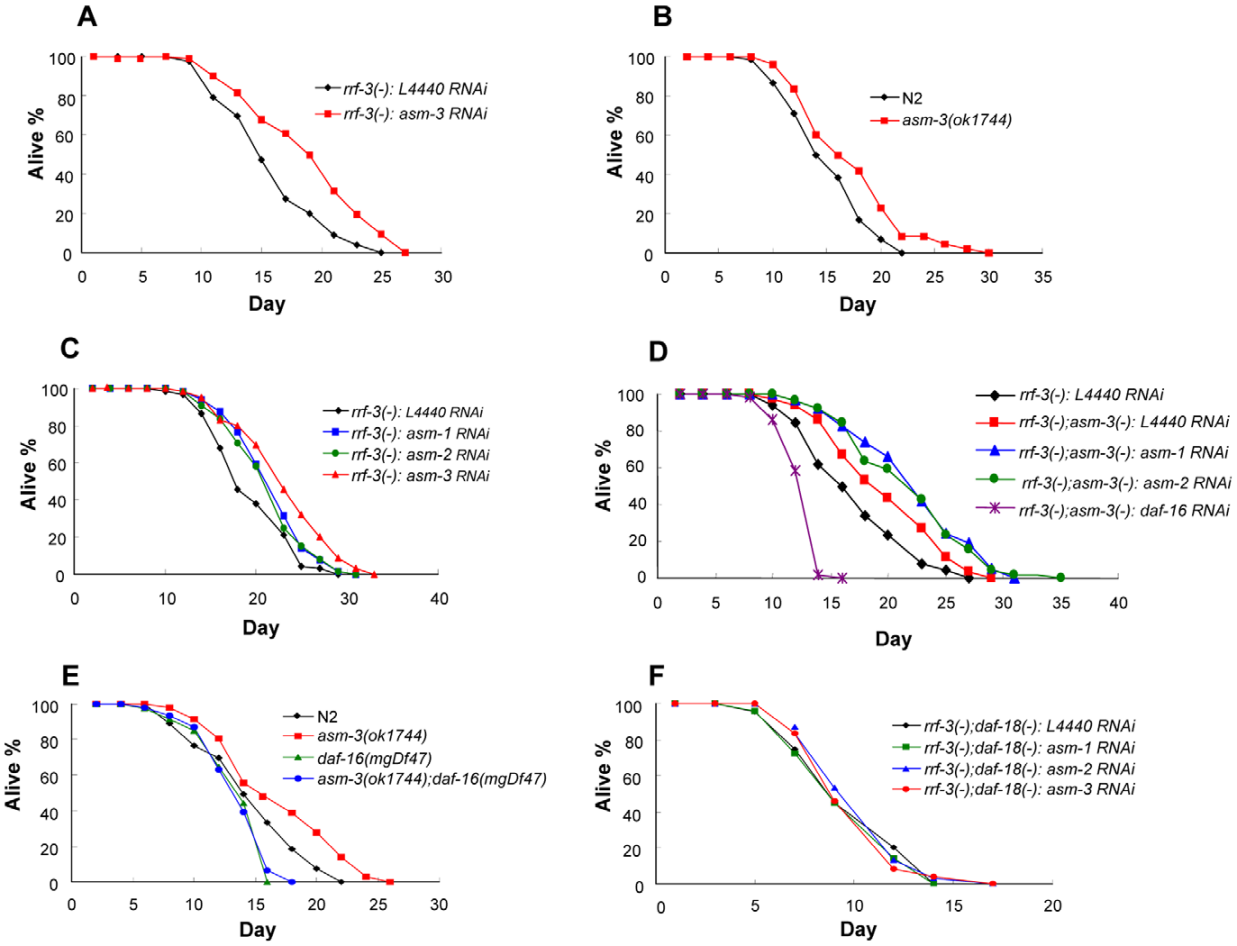
Loss of *asm* gene activities extends animal lifespan in a *daf-16* or *daf-18* dependent manner. For RNAi experiments, the vector alone (L4440) was used as a control. (A) *asm-3* RNAi extended animal lifespan with the mean lifespan 19% greater than that of the control in the *rrf-3(pk1426)* background (P<0.0001). (B) *asm-3(ok1744)* mutants had 14% longer lifespan than wild-type (N2) animals (P = 0.0141). (C) Knockdown of *asm-1*, *asm-2* or *asm-3* by RNAi each extended lifespan with the mean lifespan 12%, 10% or 19% greater than that of the vector control in the *rrf-3(pk1426)* background, respectively (P = 0.0068 for *asm-1* RNAi, P = 0.0258 for *asm-2* RNAi and P<0.0001 for *asm-3* RNAi). (D) Experiments were carried out in the *asm-3(+);rrf-3(pk1426)* or *asm-3(ok1744);rrf-3(pk1426)* background. *asm-3(ok1744*) mutation extended lifespan with the mean lifespan 15% greater than that of the control (P = 0.0018), and the lifespan of *asm-3(ok1744)* mutant was further enhanced by RNAi of *asm-1* or *asm-2* with the mean lifespan 30% or 28% greater than that of the control (P<0.0001 for *asm-1* RNAi or *asm-2* RNAi). Lifespan extension produced by *asm-3* mutation was inhibited by *daf-16* RNAi (P<0.0001). (E) *daf-16(mgDf47)* null mutation completely abolished lifespan extension phenotype of *asm-3* mutants. (F) Lifespan extension phenotype by *asm-1*, *asm-2* or *asm-3* RNAi shown in (C) was completely abolished by *daf-18(nr2037)* null mutation in the *rrf-3(pk1426);daf-18(nr2037)* background. Mean lifespan, P values and other details for these experiments are listed in Table 1 and Table 2.

The *C. elegans* genome encodes three ASM homologs, *asm-1*, *asm-2* and *asm-3*. Among them, ASM-3 is most closely related to human ASM, and the two proteins share 42% identity in the predicted C-terminal catalytic domain (Figure S1). The *asm-3* gene was initially discovered in our recent genome-wide RNAi screen for new genes regulating aging [25]. In the current study, we found that RNAi-mediated inactivation of *asm-3* produces a lifespan extension phenotype (Figure 1A). The mean lifespan of animals treated with *asm-3* RNAi was 19% longer than that of the vector control (Table 1, Set #1). To verify lifespan extension phenotype in *asm-3* mutant, we also investigated a chromosomal mutation of *asm-3*, *asm-3(ok1744)*, a putative null allele with a 1558 bp deletion and a 7 bp insertion in the predicted catalytic domain (Figure S2). Consistent with RNAi experiments, the *asm-3(ok1744)* mutants also extended animal lifespan by 14% as compared to the wild-type (Figure 1B; Table 2, Set #1). In *C. elegans,* two other paralogs, *asm-1* and *asm-2,* encode polypeptides highly homologous to ASM-3 (Figure S3). We thus examined their roles in lifespan regulation. When *asm-1* or *asm-2* was inactivated by RNAi knockdown, a modest lifespan extension phenotype was observed (Figure 1C), with the mean lifespan 12% or 10% greater than that of the control, respectively (Table 1, Set #2). We speculated that there may be functional redundancy between *asm-3*, *asm-1* and *asm-2*. Indeed, double inactivation of *asm-3* and *asm-1* or of *asm-3* and *asm-2* further extended lifespan, with the mean lifespan 30% or 28% greater than that of the control, respectively (Figure 1D; Table 1, Set #3). These results suggest that *asm-3*, *asm-1* and *asm-2* each contributes to the regulation of animal lifespan, and inactivating two *asm* genes produces an additive effect on lifespan extension. Together, our results demonstrate the importance of the *asm* gene family in the regulation of lifespan and highlight *asm-3* as the most prominent member in this process.

**Table 1:** Summary of adult lifespan assays after RNAi-mediated gene inactivation.

Genotype	RNAi	MeanLifespan ± SEM(Days)	Relative MeanLifespan (%)	*Relative MeanLifespan (%)	P value	* P value
Set #1						
*rrf-3(pk1426)*	L4440	16.1±0.47	100	–	–	–
	*asm-3*	19.1±0.59	119	–	<0.0001	–
Set #2						
*rrf-3(pk1426)*	L4440	19.6±0.54	100	–	–	–
	*asm-1*	22.0±0.52	112	–	0.0068	–
	*asm-2*	21.6±0.57	110	–	0.0258	–
	*asm-3*	23.4±0.66	119	–	<0.0001	–
Set #3						
*rrf-3(pk1426)*	L4440	17.4±0.57	100	–	–	
*asm-3(ok1744);rrf-3(pk1426)*	*L4440	20.1±0.61	115	100	0.0018	–
	*asm-1*	22.7±0.64	130	113	<0.0001	0.0019
	*asm-2*	22.3±0.65	128	111	<0.0001	0.0072
	*daf-16*	12.8±0.20	–	64	–	<0.0001
Set #4						
*rrf-3(pk1426);daf-18(nr2037)*	L4440	9.9±0.60	100	–	–	–
	*asm-1*	10.0±0.55	103	–	0.7936	–
	*asm-2*	10.4±0.46	106	–	0.6009	–
	*asm-3*	10.1±0.49	102	–	0.9618	–
Set #5						
*aap-1(m889)*	L4440	42.41±1.79	100	–	–	–
	*asm-3*	51.33±2.23	121	–	<0.0001	–

RNAi treatments, initiated at L1 stage, were continued throughout the assay time. Lifespan assays were performed at 20°C. Each experiment was individually grouped and statistical analyses of the data sets were carried out using the vector (L4440) control as the reference in each group (P value). In Set #3, the second set of statistical analyses was carried out for all the survivor data derived from the *asm-3(ok1744);rrf-3(pk1426)* strain, using the corresponding vector (L4440) control in this strain (marked as *) as the reference for this subgroup (*P values). Each set of the lifespan experiments was repeated at least two independent times and similar results were obtained. Data from representative sets of experiments are shown. Greater than 50 worms were counted for each RNAi-inducing condition in each experiment.

**Table 2:** Summary of adult lifespan assays in various mutant backgrounds.

Genotype	Mean Lifespan ± SEM (Days)	Relative Mean Lifespan (%)	P value	* P value
Set #1				
N2 (wild-type)	15.3±0.58	100	–	–
*asm-3(ok1744)*	17.4±0.69	114	0.0141	–
Set #2				
N2 (wild-type)	14.8±0.57	100	–	–
*asm-3(ok1744)*	17.1±0.80	116	0.0077	–
*daf-16(mgDf47)	13.6±0.47	92	0.0303	–
*asm-3(ok1744);daf-16(mgDf47)*	13.7±0.4	93	0.0165	0.8974
Set #3				
N2 (wild-type)	16.2±0.56	100	–	–
*asm-3(ok1744)*	18.7±0.80	116	0.005	–
*daf-2(e1370)	39.4±1.34	244	<0.0001	–
*asm-3(ok1744);daf-2(e1370)*	40.1±1.51	248	<0.0001	0.463
Set #4 (FuDR)				
N2 (wild-type)	19.4±0.22	100	–	–
*asm-3(ok1744)*	21.2±0.49	109	0.0005	–
*age-1(mg305)	41.7±0.98	215	<0.0001	–
*asm-3(ok1744);age-1(mg305)*	69.6±1.52	359	<0.0001	<0.0001
Set #5				
N2 (wild-type)	14.4±0.46	100	–	–
*asm-3(ok1744)*	16.7±0.64	116	0.0056	–
*pdk-1(sa709)	21.1±1.25	147	<0.0001	–
*asm-3(ok1744);pdk-1(sa709)*	21.7±1.07	151	<0.0001	0.8404
Set #6				
N2 (wild-type)	15.7±0.56	100	–	–
*asm-3(ok1744)*	18.1±0.87	115	0.0039	–
*akt-1(mg306)	21.8±0.83	139	<0.0001	–
*asm-3(ok1744);akt-1(mg306)*	14.8±0.81	95	0.8422	<0.0001

All the assays were carried out at 20°C and on regular NGM plates. For Set #4, assays were carried out on plates containing FuDR (50 µg/ml) to avoid the internal hatching of the *asm-3(ok1744);age-1(mg305)* mutant animals. Each experiment was individually grouped and statistical analyses were carried out for the experiment data set and the wild-type control data set in each group (P values). In addition, statistical analyses were carried out for the survival data of the assayed single mutant (marked as *) and the corresponding double mutant containing the *asm-3(ok1744)* allele (*P values). In Set #6, P value between *asm-3(ok1744)* and *asm-3(ok1744);akt-1(mg306)* is 0.064. Each set of the lifespan experiments was repeated at least two independent times and similar results were obtained. Data from representative sets of experiments are shown. Greater than 50 worms were counted for each strain in each experiment.

### Genetic Interactions of *asm-3* with Negative Regulators of the *daf-2*/IIS Pathway

To investigate whether *asm-3* functions in the *daf-2*/IIS pathway, we examined genetic interactions of *asm-3* with *daf-16* and *daf-18*, which negatively regulate the *daf-2*/IIS pathway. Inactivation of *daf-2* dramatically extends animal lifespan, and this effect can be completely suppressed by loss-of-function *daf-16* mutations [1]. We found that *daf-16* RNAi abolished the lifespan extension phenotype caused by *asm-3(ok1744)* mutation (Figure 1D; Table 1, Set #3). To verify this result, we constructed double mutant strain carrying both *asm-3(ok1744)* and *daf-16(mgDf47)* null alleles. We found that the lifespan extension phenotype of the *asm-3(ok1744)* mutants was completely suppressed by the null mutation of *daf-16* (Figure 1E; Table 2, Set #2).

On the other hand, inactivation of *daf-18*, encoding *C. elegans* homolog of human PTEN tumor suppressor, is known to suppress lifespan extension and constitutive dauer arrest caused by deficiency in *daf-2* or *age-1* [14], [15]. To test the epistatic relationship between *asm-3* and *daf-18,* we first attempted to construct a double mutant strain carrying both *asm-3(ok1744)* and *daf-18(nr2037)* alleles but our effort failed, as these two alleles are located in close proximity on the same chromosome IV region. Instead, we have carried out RNAi experiments in the *rrf-3(pk1426);daf-18(nr2037)* mutant strain for the epistatic analysis of *asm-3* and *daf-18*. The presence of the *rrf-3(pk1426)* allele is to enhance the RNAi efficiency. Consequently, we observed that the lifespan extension phenotypes produced by inactivation of *asm-1, asm-2* or *asm-3* gene (Figure 1C; Table 1, Set #2) were all suppressed by a null mutation of *daf-18*, *daf-18(nr2037)* (Figure 1F; Table 1, Set #4). Thus, these results show that *asm-3* behaves similarly to *daf-2* in that the lifespan extension phenotypes caused by *asm-3* or *daf-2* loss-of-function mutations require *daf-16* and *daf-18* gene activity.

### Genetic Interactions of *asm-3* with Major Components of the *daf-2*/IIS Pathway

**Figure 2:**
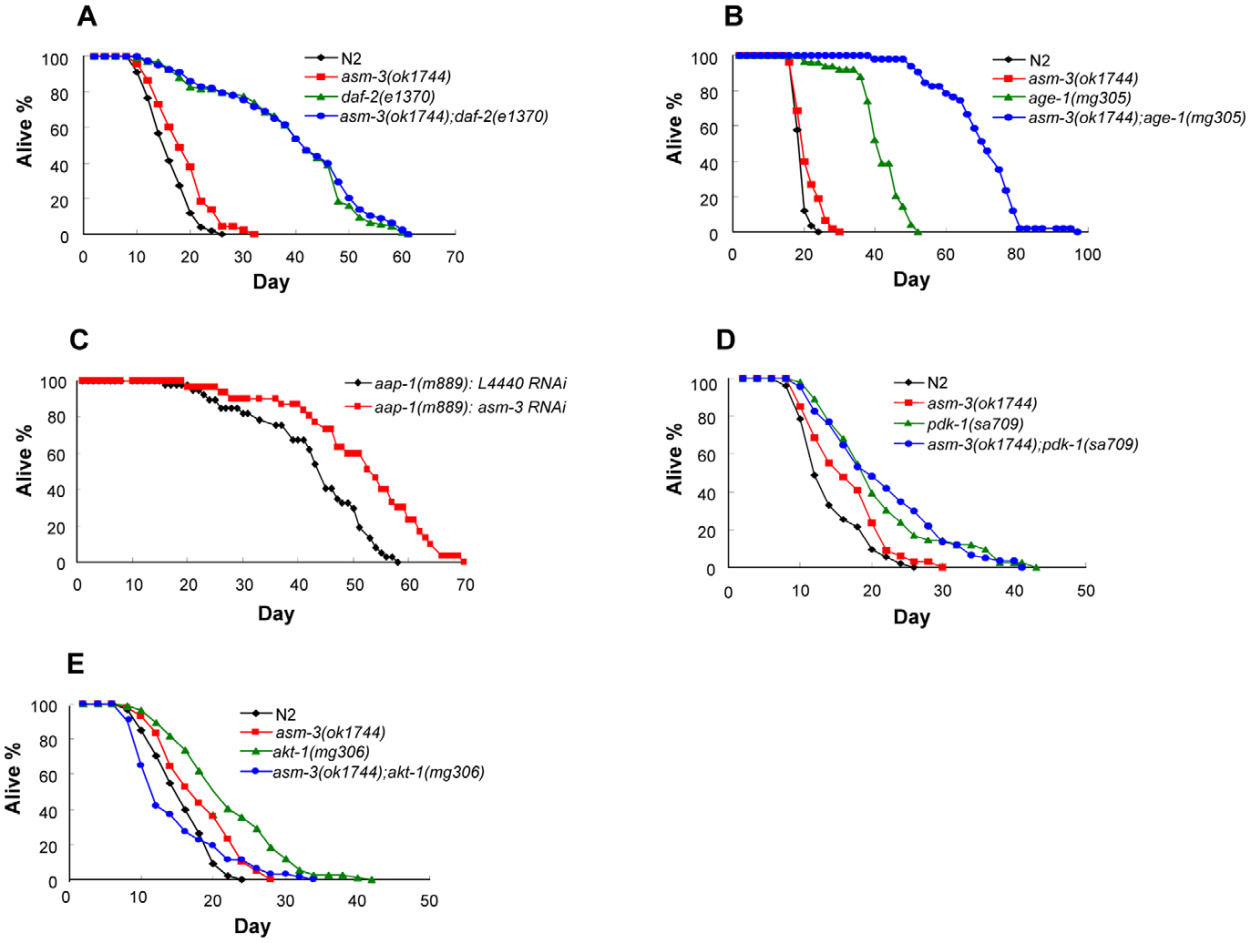
Effects of *asm-3* on lifespan regulation in various mutants defective in the *daf-2* signaling. (A) Loss of *asm-3* did not further increase the lifespan of *daf-2(e1370)* mutants (P = 0.463). (B) *asm-3(ok1744)* mutation enhanced the mean lifespan of the longer-lived *age-1(mg305)* mutants by 67% (P<0.0001). (C) Silencing of *asm-3* in the *aap-1(m889)* mutant background further extended the mean lifespan by 21% compared to control (L4440) RNAi (P<0.0001). (D) *asm-3* mutation did not affect lifespan of *pdk-1(sa709)* mutant animals (P = 0.8404). (E) Effects of *asm-3(ok1744), akt-1(mg306),* and *asm-3(ok1744);akt-1(mg306)* mutations on lifespan regulation. *asm-3* mutation inhibited the lifespan extension of *akt-1(mg306)* mutant (P<0.0001), but *akt-1* mutation did not seem to affect the lifespan extension of *asm-3(ok1744)* mutant (P = 0.064). Mean lifespan, P values and other details for these experiments are listed in Table 1 and Table 2.

To investigate how *asm-3* regulates lifespan in the *daf-2*/IIS pathway, we examined genetic interactions between *asm-3* and major components of the *daf-2*/IIS pathway. While partial loss-of-function *daf-2(e1370)* mutants had dramatically extended lifespan at 20°C [1], we found that the lifespan of *daf-2* mutants was not further extended by the loss of *asm-3* (Figure 2A; Table 2, Set #3). In the *asm-3(ok1744);daf-2(e1370)* double mutant, it is possible that *daf-2* signaling was reduced to below threshold levels by the *daf-2(e1370)* mutation, and thus the *asm-3(ok1744)* mutation could not further dampen the *daf-2* signaling outputs. Thus, this result suggests that *asm-3* and *daf-2* function in the same pathway. We also examined genetic interaction between *asm-3* and *age-1*. The *age-1* gene encodes a homolog of mammalian PI-3 kinase catalytic subunit [26]. Partial loss-of-function *age-1(mg305)* mutants have a dramatically extended lifespan phenotype [27]. Remarkably, our study showed that *asm-3(ok1744);age-1(mg305)* double mutants had mean lifespan 67% greater than that of *age-1(mg305)* single mutants, or 259% greater than that of wild-type animals (Figure 2B; Table 2, Set #4). Consistently, we also observed a cooperative effect on animal lifespan extension when *asm-3* was inactivated in another partial loss-of-function *age-1* mutant, *age-1(hx546)* [26] (data not shown). Subsequently, we analyzed the genetic interaction between *asm-3* and *aap-1*. It has been previously reported that *aap-1,* encoding a *C. elegans* homolog of mammalian p85 regulatory subunit of PI-3 kinase, functions in the same pathway as *age-1* [28]. While partial loss-of-function *aap-1(m889)* mutants exhibited longer lifespan phenotype than wild-type animals, silencing of *asm-3* in the *aap-1(m889)* mutants further extended the mean lifespan by 21% (Figure 2C; Table 1, Set #5). These genetic results indicate that loss of *asm-3* cooperates with an *age-1* or *aap-1* loss-of-function mutation to extend animal lifespan, suggesting that *asm-3* potentiates *age-1/aap-1* signaling. We also tested *asm-3* interaction with *pdk-1*, which encodes a kinase that acts downstream of AGE-1 but upstream AKT-1/2 in the DAF-2/IIS pathway [29]. Loss of *asm-3* did not further extend the lifespan in the partial loss-of-function *pdk-1(sa709)* [29] mutant background (Figure 2D; Table 2, Set #5). This result suggests that *asm-3* and *pdk-1* likely function in the same pathway. In addition, we tested the genetic interaction of *asm-3(ok1744)* with a null mutation of *akt-1*, *akt-1(mg306)* [30]. We observed that the *asm-3(ok1744)* mutation shortened the lifespan of the longer lived *akt-1(mg306)* mutant animals, but the *akt-*1*(mg306)* mutation did not seem to affect the lifespan extension phenotype of the *asm-3(ok1744)* mutant (Figure 2E; Table 2, Set #6). The fact that the *asm-3(ok1744)* mutation can suppress the lifespan extension phenotype of the *akt-1(mg306)* mutant suggests that there may be additional genetic interactions between the gene families of *akt* and *asm*. Taken together, these results show that *asm-3* plays an important role in the *daf-2*/IIS pathway to regulate animal lifespan.

### Loss of *asm-3* Results in an Increased Resistance to Environmental Stress

Many long-lived mutants of the *daf-2*/IIS pathway have strong resistance toward environmental stress such as oxidative stress [4], [31]–[35] and heat stress stimuli [33], [34], [36], [37] and this response is regulated by DAF-16/FOXO [31]–[35]. Thus, we tested whether *asm-3* could regulate stress response under the oxidative stress or heat stress condition. We used paraquat, a chemical to produce reactive oxygen species *in vivo*, for the oxidative stress test and high temperature (35°C) for the heat stress response, as reported previously [31]–[37]. We found that *asm-3(ok1744)* mutant adult worms had increased resistance towards paraquat as compared to the wild-type animals (Figure S4A). In addition, the *asm-3(ok1744)* mutant adult animals were more resistant against heat stress of 35°C when compared to wild-type animals (Figure S4B). From those two stress response assays, we also found that *daf-16(mgDf47)* mutants were very sensitive to environmental stress responses, and the stress resistance phenotypes of the *asm-3(ok1744)* mutant was *daf-16*-dependent (Figure S4A, S4B). Thus, these data suggest that *asm-3* plays an important role in regulation of stress response, similar to the reports of enhanced resistance observed in the *daf-2(e1370)*, *age-1(hx546)* or *age-1(mg305)* mutants [31]–[34], [36], [37].

### *asm-3* Regulates Dauer Arrest by Modulating the *daf-2*/IIS Pathway

**Figure 3:**
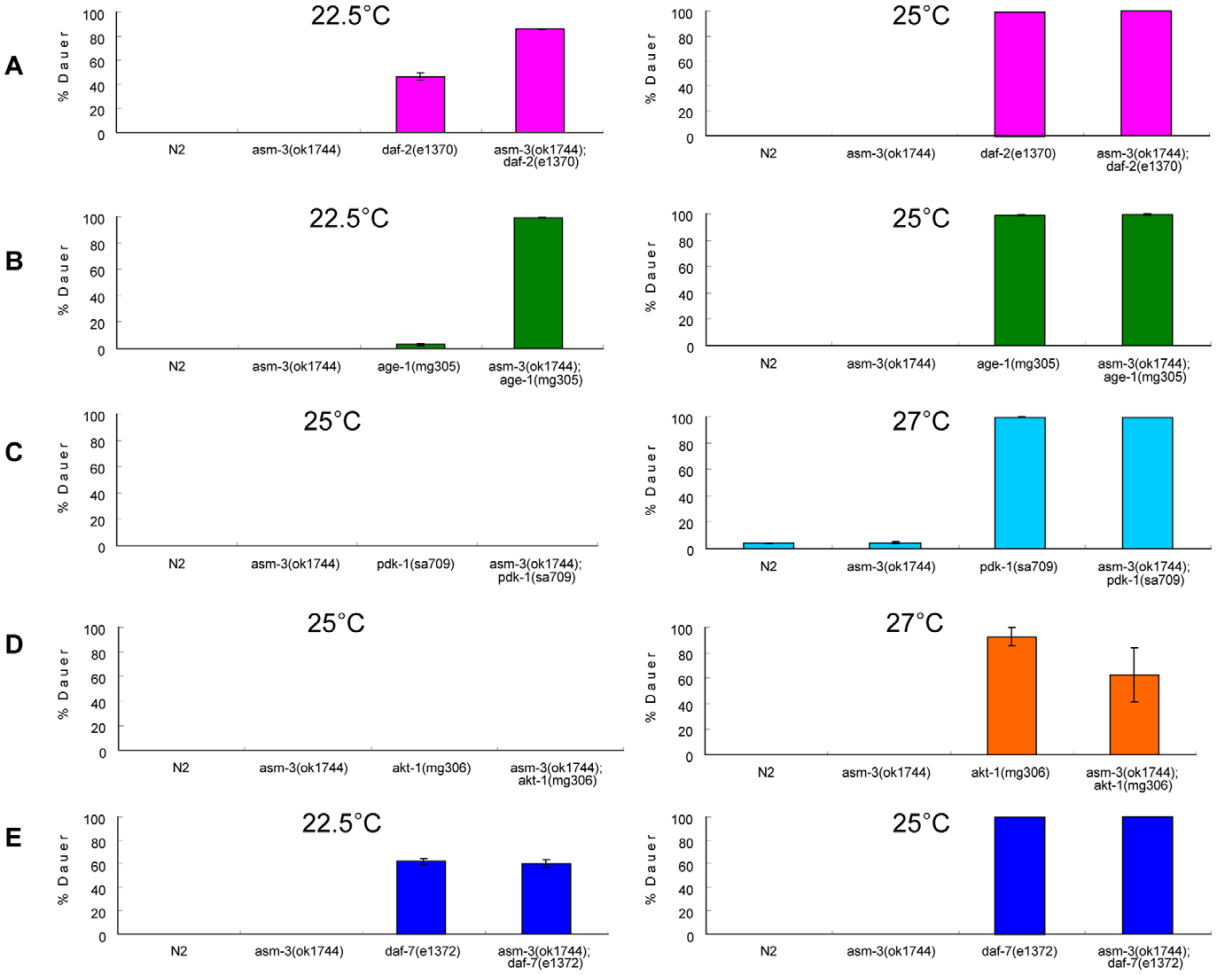
Effects of *asm-3* on dauer formation regulation in various mutants defective in the*daf-2* signaling. (A) Loss of *asm-3* enhanced dauer formation of *daf-2(e1370)* mutants at the semi-permissive temperature 22.5°C. (B) *asm-3* mutation greatly enhanced dauer arrest phenotype of *age-1(mg305)* mutants at 22.5°C. (C) *asm-3* mutation did not affect dauer arrest induced by the *pdk-1(sa709)* mutation at 27°C. The mutant animals carrying *sa709* allele formed dauer at 27°C but not at 25°C. (D) *asm-3* mutation partially suppressed the dauer arrest phenotype of *akt-1(mg306)* mutants at 27°C. No dauers at 25°C were observed for the *akt-1(mg306)* mutant animals with or without the presence of the *asm-3(ok1744)* allele. (E) *asm-3* mutation had no effect on dauer arrest phenotype of *daf-7(e1372)* mutants at either 22.5°C or 25°C. The *asm-3(ok1744)* allele by itself did not induce dauer formation at either 22.5°C or 25°C. Error bars indicate standard deviation from triplicates. Details including total worm numbers used in the assay are listed in Table S1.

In *C. elegans*, dauer arrest is controlled by a variety of signal transduction pathway [3], [10]. Dauer, a hibernation-like state, is normally induced by starvation, high population density or high temperature [3], [10]. Mutants with reduced signaling in the *daf-2*/IIS pathway form dauer even under favorable growth conditions [3], [10]. We investigated whether *asm-3* participates in the *daf-2/*IIS pathway to regulate dauer formation. As previously reported [28], [38], [39], temperature-sensitive *daf-2(e1370)* mutants are prone to induction of dauer arrest at 25°C and less so at 22.5°C. Consistent with previous reports, our results showed that the *daf-2(e1370)* mutants formed dauer constitutively at non-permissive temperature 25°C, but formed dauer less efficiently at semi-permissive 22.5°C (Figure 3A; Table S1). However, the dauer arrest at 22.5°C in *daf-2(e1370)* mutants was markedly enhanced from 46% to 86% by the presence of the *asm-3(ok1744)* allele (Figure 3A; Table S1). In addition, *age-1(mg305)* mutants possessed a constitutive dauer phenotype at 25°C [27] but rarely displayed dauer arrest phenotype at 22.5°C (Figure 3B; Table S1). However, loss of *asm-3* dramatically enhanced dauer arrest of *age-1(mg305)* mutants at 22.5°C from 3% to 99% (Figure 3B; Table S1). On the other hand, loss of *asm-3* by itself did not induce dauer arrest at 25°C (Figure 3A, 3B; Table S1). Therefore, these data indicate that the *asm-3* gene activity potentiates *daf-2* and *age-1* signaling to regulate dauer arrest. In comparison, because dauer formation phenotype of the *pdk-1(sa709)* mutant was so penetrant at 27°C [29] (Figure 3C; Table S1), we could not detect any effects of *asm-3(ok1744)* mutation on *pdk-1(sa709)* at this temperature (Figure 3C; Table S1). Loss of *asm-3* did not trigger dauer formation in *pdk-1(sa709)* mutants at 25°C (Figure 3C; Table S1). However, for *akt-1(mg306)* null mutants, which were shown to form dauer efficiently at 27°C but not at 25°C [30], [40], loss of *asm-3* partially suppressed dauer formation of *akt-1(mg306)* mutants at 27°C (Figure 3D; Table S1). The genetic interactions of *asm-3* with *pdk-1* or *akt-1* in dauer formation appear to be consistent with those in lifespan regulation (Figure 3C, 2D; Figure 3D, 2E). Taken together, our genetic analyses suggest that *asm-3* acts in the *daf-2*/IIS pathway to regulate dauer formation.

In addition to the *daf-2*/IIS pathway, the *daf-7*/TGF-β-like signaling pathway is also involved in the regulation of dauer formation. The *daf-7* gene encodes a ligand related to mammalian transforming growth factor beta (TGF-β) [41]. The *daf-7(e1372)* mutants are known to constitutively form dauer at 25°C [41]. We found that, at either 25°C or 22.5°C, loss of *asm-3* did not affect the constitutive dauer arrest phenotype of *daf-7(e1372)* mutants (Figure 3E; Table S1). These results thus indicate that dauer regulation via the *daf-7*/TGF-β signaling pathway does not require *asm-3* gene activity. These results suggest that the involvement of *asm-3* in the *daf-2*/IIS pathway is specific.Loss of *asm* Genes Results in Nuclear Localization of DAF-16

**Figure 4:**
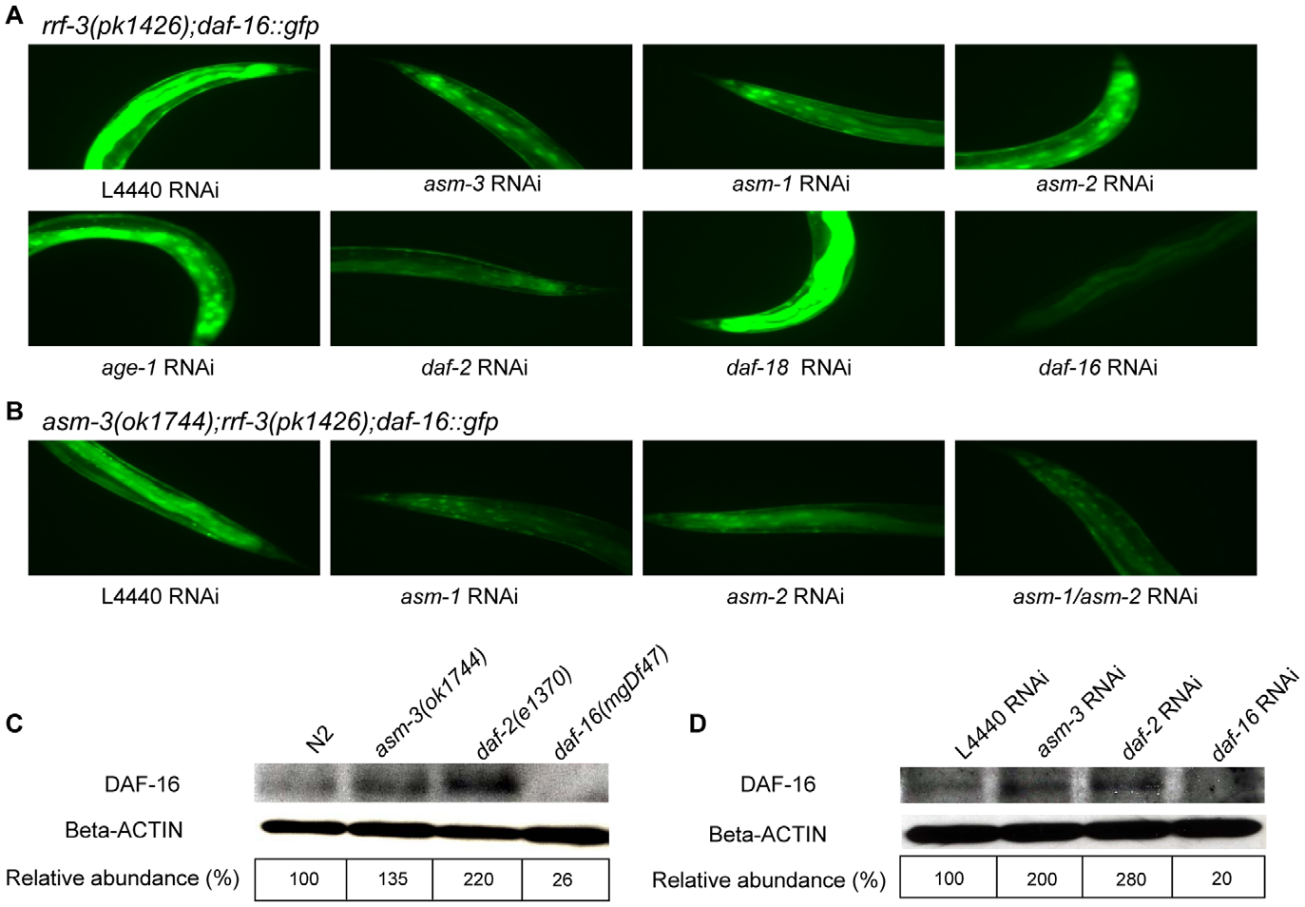
Loss of *asm* induces the nuclear localization of DAF-16::GFP fusion protein and affects the DAF-16 protein levels. (A) and (B) DAF-16::GFP cellular distributions were examined by fluorescence microscopy and tail regions of animals were shown here. For images on the body and head regions of the animals, see Figure S5. Animals were examined on adult day 1 (A) or day 4 (B). (A) In the *rrf-3(pk1426);daf-16::gfp* mutant background, vector control (L4440) RNAi showed that DAF-16::GFP diffusely localized in the cytoplasm, while *asm-3*, *asm-1* or *asm-2* RNAi each induced the nuclear localization of DAF-16::GFP. RNAi inactivation of *daf-2* and *age-1* (positive controls) and RNAi inactivation of *daf-16* and *daf-18* (negative controls) were carried out in parallel. (B) In the *asm-3(ok1744);rrf-3(pk1426);daf-16::gfp* mutant background, RNAi knockdown of *asm-1*, *asm-2* or *asm-1/asm-2* (double RNAi of *asm-1* and *asm-2*) further induced the nuclear localization of DAF-16::GFP protein. (C) and (D) western blot analysis of endogenous DAF-16 protein levels. (C) Increased DAF-16 protein levels were observed in the *asm-3(ok1744)* and *daf-2(e1370)* mutants as compared with that of wild-type control. Lysates were prepared from adult day 1 animals. (D) RNAi knockdown of *asm-3* or *daf-2* each elevated DAF-16 protein level as compared with that of vector control (L4440) RNAi. The specificity of the immunodetection was verified by the disappearance of DAF-16 protein in the *daf-16(mgDf47)* null mutants or in animals treated with *daf-16* RNAi. Lysates were prepared from RNAi-treated, adult day 2 animals. In (C) and (D), quantification of the relative abundance of DAF-16 proteins was shown with the DAF-16 protein levels being normalized against the beta-actin protein levels using the ImageJ software.

In live animals, the downstream signaling output of the DAF-2/IIS pathway can be assayed by the intracellular localization of DAF-16/FOXO. DAF-16 is normally sequestered in the cytoplasm after phosphorylation by AKT-1/AKT-2, while mutations causing reduced signaling in the *daf-2* pathway all lead to translocation of DAF-16 to the nucleus [3], [9]. To further investigate whether *asm-3* directly regulates the molecular signaling cascade of DAF-2 to DAF-16, we used a strain carrying a *daf-16::gfp* transgene to monitor the intracellular distribution of DAF-16::GFP fusion protein [9]. We constructed mutant strains of *rrf-3(pk1426);daf-16::gfp* and *asm-3(ok1744);rrf-3(pk1426);daf-16::gfp* with the presence of the *rrf-3(pk1426)* allele to enhance the RNAi efficiency. We found that inactivation of individual *asm* genes each induced nuclear localization of DAF-16::GFP in the *rrf-3(pk1426);daf-16::gfp* background (Figure 4A; Figure S5). As expected, *age-1*, *daf-2*, or *akt-1* RNAi, which were used as positive controls, all resulted in the nuclear localization of DAF-16::GFP (Figure 4A; Figure S5). In contrast, control (L4440) RNAi and *daf-18* RNAi led to a diffused distribution of DAF-16::GFP in cytoplasm, while *daf-16* RNAi greatly decreased fluorescence intensity of DAF-16::GFP (Figure 4A; Figure S5). We also tested the effects of multiple knockdowns of *asm* genes. We found that combined inactivation of 2 or 3 *asm* genes caused a further increase in the nuclear localization of DAF-16::GFP in the *asm-3(ok1744);rrf-3(pk1426);daf-16::gfp* mutant background (Figure 4B; Figure S6). In addition, the nuclear translocation of DAF-16::GFP in the *asm-3(ok1744)* mutant was abolished by *daf-16* RNAi or *daf-18* RNAi (Figure S6). These results are consistent with the observations that *daf-16* and *daf-18* gene activities are required for the *asm*-mediated lifespan regulation (Figure 1D-1F). Taken together, these results indicate that *asm-3* regulates DAF-16 through controlling its intracellular localization.

### Loss of *asm-3* Increases Endogenous DAF-16 Protein Expression

The activity and protein levels of the DAF-16/FOXO transcription factor are essential for the lifespan regulation in the *daf-2*/IIS pathway [11]–[13]. Increased protein levels of endogenous DAF-16 have been reported for the longer-lived *rle-1* mutants [12] or *eak-7* mutant [42], and overexpression of the DAF-16 homolog in *Drosophila* leads to extension of lifespan [43], [44]. We therefore examined the DAF-16 protein levels in animals where the *asm-3* gene was inactivated. We observed that endogenous DAF-16 protein levels were elevated by the *asm-3(ok1744)* mutation or by *asm-3* RNAi (Figure 4C, 4D; an increase of 35% or 100%, respectively). Interestingly, we also observed that either *daf-2(e1370)* mutation or *daf-2* RNAi led to a significant increase in DAF-16 protein levels (Figure 4C, 4D; an increase of 120% or 180%, respectively). As controls, wild-type animals or animals treated with vector (L4440) RNAi were used, respectively. Taken together, our results indicate that ASM-3 and DAF-2 each negatively regulates the endogenous DAF-16 protein expression levels. The increased DAF-16 protein levels, in addition to the nuclear translocation of DAF-16, may constitute a signaling output from the *daf-2*/IIS pathway.

### Loss of *asm-3* Promotes the Expression of *daf-16* Target Genes

**Figure 5:**
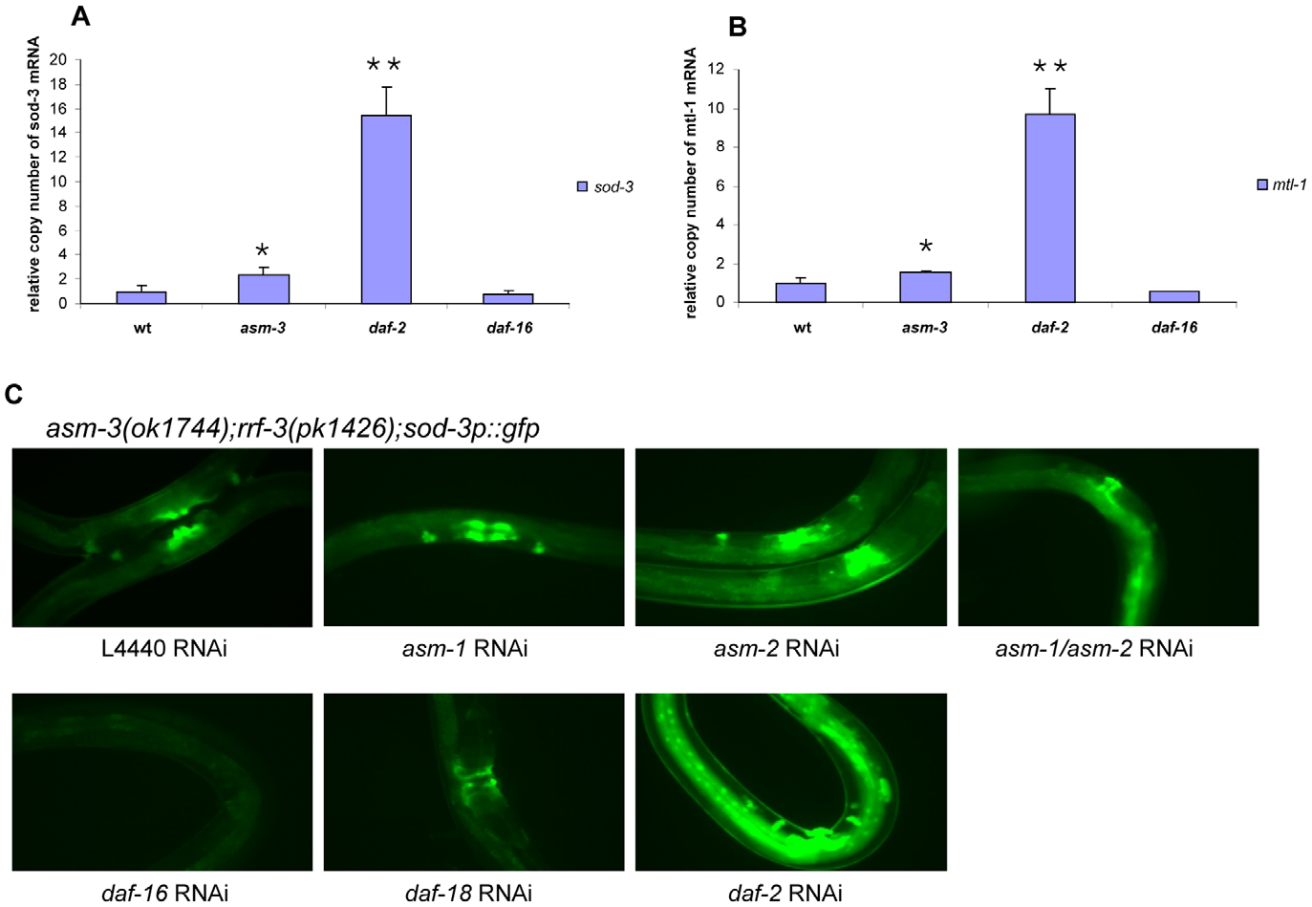
qRT-PCR for DAF-16/FOXO transcriptional activity and *sod-3p::gfp* reporter. (A) By qRT-PCR, mRNA expression of endogenous *sod-3* gene was increased about 3-fold in the *asm-3(ok1744)* mutant animals as compared to the wild-type (N2) animals (T-test *P<0.05 for *asm-3(ok1744)* vs. wild-type; T-test **P<0.001 for *daf-2(e1370)* vs. wild-type). RNA samples were prepared from young adults (adult day 1). (B) By qRT-PCR, mRNA expression of endogenous *mtl-1* gene was modestly increased in the *asm-3(ok1744)* mutant animals as compared to the wild-type (N2) animals (T-test *P<0.05 for *asm-3(ok1744)* vs wild-type; T-test **P<0.001, *daf-2(e1370)* vs. wild-type). In (A) and (B), RNA samples, isolated from *daf-2(e1370)* or *daf-16(mgDf47)* mutant animals, were used as positive and negative controls, respectively. Additionally, an internal control of *act-1* was used for qRT-PCR and relative mRNA expression levels of *sod-3* or *mtl-1* were normalized to that of *act-1*. (C) Increased SOD-3::GFP expression was observed when multiple *asm* genes were inactivated in the *asm-3(ok1744);rrf-3(pk1426);sod-3p::gfp* mutant background compared to the vector control (L4440) RNAi. As negative controls, RNAi of *daf-16* or *daf-18* was used. As a positive control, the *daf-2* RNAi was used. Animals, treated with the indicated RNAi molecules, were examined on adult day 3. All fluorescence microscopy images were photographed using identical exposure times.

As described above, inactivation of *asm-3* triggers nuclear translocation of DAF-16/FOXO and up-regulates endogenous DAF-16 protein levels. However, as previously reported [45]–[47], nuclear translocation of DAF-16/FOXO is not sufficient for activation of the DAF-16/FOXO transcription activity. We therefore examined whether loss of *asm-3* indeed results in an increase of activity of *daf-16*/FOXO transcription factor, by analyzing the expression levels of several known DAF-16/FOXO target genes [48], [49]. The *sod-3* gene, encoding superoxide dismutase, is directly regulated by DAF-16; and in partial loss-of-function *daf-2* mutants, a GFP transgene under control of the *sod-3* promoter (*sod-3p::gfp*), is highly expressed [11]. We therefore examined transcriptional expression of endogenous *sod-3* by using qRT-PCR analysis. We observed that *sod-3* mRNA levels were increased by about 3-fold in *asm-3(ok1744)* mutants over the wild-type (Figure 5A). The expression of another DAF-16-regulated gene, *mtl-1* [48], encoding metallothioneins, was also modestly increased in *asm-3* mutants (Figure 5B). These results indicate that *asm-3* regulates expression of *daf-16* target genes through controlling DAF-16/FOXO transcriptional activity. In addition, we used a strain carrying the *sod-3p::gfp* transgene to monitor the *daf-16*/FOXO transcriptional activity. Inactivation of a single *asm* gene has only a modest effect on the *sod-3p:gfp* reporter gene expression (data not shown). However, inactivation of 2 or 3 *asm* genes, achieved through RNAi of *asm-1, asm-2* or *asm-1* and *asm-2* together in the *asm-3(ok1744);rrf-3(pk1426);sod-3p::gfp* mutant background, each led to a marked up-regulation of the *sod-3p::gfp* expression as compared to control vector (L4440) RNAi (Figure 5C). As negative controls, *daf-16* RNAi and *daf-18* RNAi each caused a dramatic reduction of the *sod-3p::gfp* expression when assayed in parallel (Figure 5C). These data indicate that *asm* genes regulate the transcriptional output of *daf-16*.

### Chemical Inhibitors of ASM Delay Aging in *C. elegans*

**Figure 6:**
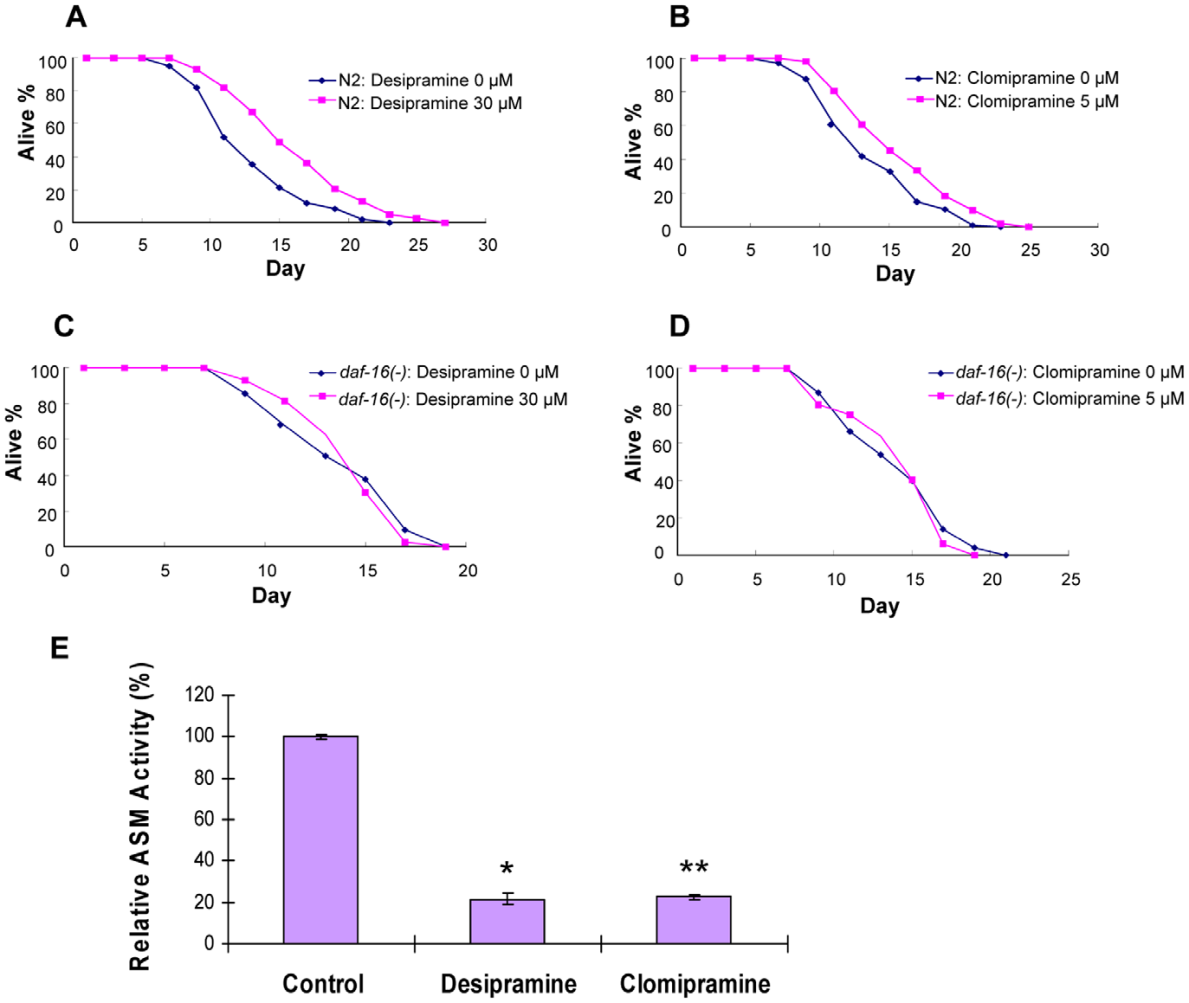
Effects of ASM inhibitors on lifespan regulation and enzyme activity in *C. elegans*. Lifespan assays were carried out at 20°C. (A) and (B) Wild-type (N2) animals were plated on plates containing 30 µM desipramine (A), 5 µM clomipramine (B) or vehicle control (0 µM in panel A or B). A significant lifespan extension was observed in either desipramine-treated or clomipramine-treated wild-type animals as compared to vehicle control wild-type animals (24% increase and P<0.0001 for desipramine-treated wild-type animal; 14% increase and P = 0.0012 for clomipramine-treated wild-type animals). (C) and (D) The *daf-16(mgDf47)* mutant animals were plated on plates containing 30 µM desipramine (C), 5 µM clomipramine (D) or vehicle control (0 µM in panel C or D). No lifespan extension was observed in drug-treated *daf-16(mgDf47)* null mutant animals. Mean lifespan and P values for the experiments represented here are listed in Table 3. (E) Desipramine and clomipramine each inhibited CeASM activity. Synchronized L1 animals were treated with desipramine, clomipramine or vehicle control (see Material and Methods for details). Lysates were prepared from L4 animals and assays were performed in duplicates using [^14^C]-sphingomyelin. CeASM activities from all samples were normalized against that in the vehicle control (100%). Desipramine or clomipramine treatment each decreased CeASM activity by 78% or 77%, respectively (T-test *P<0.001 for desipramine and **P<0.001 clomipramine vs. vehicle control, respectively).

Our genetic analyses suggest that ASM may be a potential target for anti-aging at the organismal level. It has been reported that mammalian ASM activity can be inhibited by the chemicals desipramine and clomipramine [50]–[52], which are clinically approved drugs for anti-depression. Desipramine and clomipramine have been used for studying the role of human ASM in CD95 death receptor signaling [53], [54]. Indeed, we found that these drugs inhibited mammalian ASM activity in cultured cells (data not shown). We then tested whether desipramine and clomipramine had any effects on *C. elegans* lifespan. Remarkably, we found that treatment with desipramine (30 µM) or clomipramine (5 µM) each extended the lifespan of wild-type animals, resulting in a mean lifespan 24% or 14% greater than that of control animals, respectively (Figure 6A, 6B; Table 3, Set #1, #2). Furthermore, the effects of these drugs on lifespan extension were dependent on *daf-16* gene activity, as no lifespan extension was observed in *daf-16(mgDf47)* null mutants (Figure 6C, 6D; Table 3, Set #3, #4). These results are consistent with the data shown in Figure 1. To verify that these drugs indeed target CeASMs, we performed CeASMs activity assays using lysates prepared from drug-treated or vehicle-treated animals. We found that treatment with desipramine or clomipramine each decreased CeASM activity by 78% or 77% as compared to vehicle control, respectively (Figure 6E). This result indicates that these chemicals effectively inhibit CeASMs. Our studies thus demonstrate that inhibition of CeASM activity by chemical compounds extends animal lifespan to an extent similar to that achieved by inactivating the *asm* genes.

**Table 3:** Effects of drug treatment on animal lifespan.

Genotype	Drug Treatment	Mean Lifespan ± SEM (Days)	Relative Mean Lifespan (%)	P value
Set #1				
N2 (wild-type)	0 µM (control)	13.1±0.40	100	–
	30 µM (desipramine)	16.3±0.51	124	<0.0001
Set #2				
N2 (wild-type)	0 µM (control)	14.0±0.41	100	–
	5 µM (clomipramine)	15.9±0.40	114	0.0012
Set #3				
*daf-16(mgDf47)*	0 µM (control)	14.0±0.45	100	–
	30 µM (desipramine)	14.3±0.39	102	0.713
Set #4				
*daf-16(mgDf47)*	0 µM (control)	14.1±0.50	100	–
	5 µM (clomipramine)	14.3±0.45	101	0.8707

N2 (wild-type) or *daf-16(mgDf47)* mutant animals were assayed on plates either containing desipramine (30 µM), clomipramine (5 µM), or no drug control (0 µM). All the lifespan assays were carried out at 20°C. Mean lifespan, relative mean lifespan and statistical analyses (P values) for each assay were listed. Standard error of the mean, SEM, is included in parenthesis. Each set of the lifespan experiments was repeated at least three independent times and similar results were obtained. Data from representative sets of experiments are shown. Greater than 50 worms were counted for each condition in each experiment.

## Discussion

### *asm-3* Functions in the *daf-2*/IIS Pathway to Regulate Animal Lifespan and Dauer Formation

In this study, we have shown that *asm-3* regulates both animal lifespan and dauer formation in *C. elegans*. Further, several lines of novel evidence suggest that *asm-3* functions in the *daf-2/*IIS pathway. First, the ability of *asm-3* deficiency to extend animal lifespan is suppressed by loss-of-function mutations in either *daf-16* or *daf-18*, two well-known negative regulators of the *daf-2* pathway (Figure 1C-1F). Second, the *asm-3(ok1744)* loss-of-function allele does not further extend the lifespan of *daf-2(e1370)* or *pdk-1(sa709)* loss-of-function mutants (Figure 2A, 2D), placing *asm-3* in the *daf-2* and *pdk-1* pathway. It is possible that the *daf-2(e1370)* or *pdk-1(sa709)* mutation has already sufficiently reduced the gene activity of *daf-2* or *pdk-1* to below the threshold levels, and thus the presence of the *asm-3(ok1744)* allele in these mutants cannot further dampen *daf-2* signaling output. Third, *asm-3* deficiency strongly enhances the lifespan extension phenotype of the *age-1(mg305)* or *aap-1(m889)* mutants (Figure 2B, 2C). As *age-1(mg305)* and *aap-1(m889)* mutations are partial loss-of-function alleles, it is possible that the *daf-2* signaling strength can be further reduced in these strains by the *asm-3(ok1744)* mutation, resulting in an increase in lifespan. Fourth, inactivation of *asm-3*, either alone or in combination with inactivation of *asm-1* or *asm-2* paralog, induces the nuclear translocation of DAF-16::GFP fusion protein, in a result similar to that achieved by inactivation of *daf-2*, *age-1* or *akt-1* (Figure 4A, 4B; Figure S5, S6). Overall, these observations suggest that *asm-3* normally functions as a positive regulator of *daf-2* signaling.

The *daf-2*/IIS pathway regulates animal development, especially dauer formation, in addition to regulating lifespan. A further line of evidence that *asm-3* functions in the *daf-2*/IIS pathway comes from the observation that the *asm-3(ok1744)* mutation strongly cooperates with the *daf-2(e1370)* or *age-1(mg305)* mutation in inducing dauer formation (Figure 3A, 3B). In addition, loss of *asm-3* did not affect dauer formation of *daf-7(e1372)* mutants, which are defective in TGF-β-like signaling (Figure 3E). These observations are similar to those reported for the *aap-1* inactivation, which can enhance the dauer phenotype of *daf-2* or *age-1* mutants, but not of *daf-1*/type I TGF-β receptor-like mutants [28]. These data further support the hypothesis that *asm-3* gene activity normally potentiates *daf-2* and *age-1* signaling.

**Figure 7:**
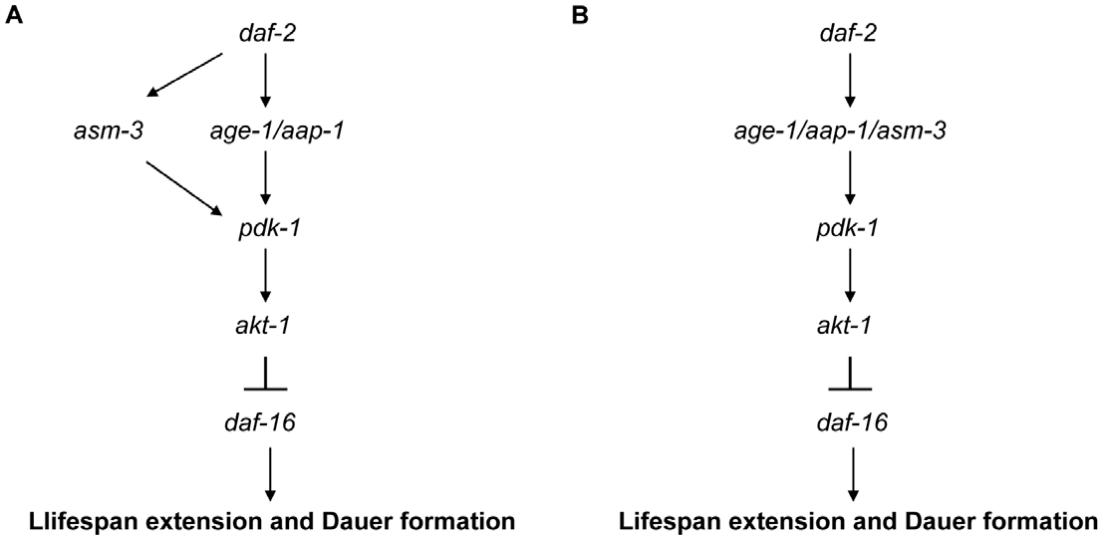
A model of *asm-3* function. *asm-3* functions downstream of *daf-2*, acting either in parallel to *age-1*/*aap-1* (A) or working together with *age-1/aap-1* (B), leading to activation of *pdk-1* and *akt-1,* and then suppression of *daf-16.*

Of particular interest are the observations that the effects of *asm-3* inactivation in *age-1* or *aap-1* mutant backgrounds are very strong. The *asm-3(ok1744)* allele can further increase the mean lifespan of the *age-1(mg305)* mutants by 67%, and *asm-3(ok1744);age-1(mg305)* double mutants have a mean lifespan 259% greater than that of wild-type animals (Figure 2B). Inactivation of *asm-3* by RNAi also potently extends the lifespan of *aap-1(m889)* mutants (Figure 2C). Consistently, *asm-3(ok1744)* enhanced the dauer formation efficiency of *age-1(mg305)* mutants from 3% to 99% at an intermediate temperature of 22.5°C (Figure 3B). Genetically, these studies suggest that *asm-3* acts in parallel to *age-1* or *aap-1*, in the *daf-2*/IIS pathway (see model in Figure 7A). Since both *age-1(mg305)* and *aap-1(m889)* are partial loss-of-function alleles, it is also possible that *asm-3* normally functions together with *age-1* or *aap-1*, through potentiating *age-1* and *aap-1* gene activities (see model in Figure 7B).

Based on our genetic data on both lifespan and dauer regulation, we propose a model in which *asm-3*, either functioning in parallel of *age-1/aap-1* (Figure 7A) or acting together with *age-1/aap-1* (Figure 7B), activates *pdk-1* and then *akt-1*, culminating in the suppression of *daf-16* to regulate animal lifespan and dauer arrest.

### *asm-3* Regulates the Localization, Transcriptional Activity, and Protein Expression Level of DAF-16/FOXO

We provide several lines of evidence suggesting that *asm-3* modulates *daf-2* signaling through affecting the DAF-16 forkhead transcription factor. We have shown that DAF-16::GFP fusion protein is translocated into the nucleus when *asm-3* is inactivated by the *asm-3(ok1744)* mutation or by RNAi (Figure 4A, 4B; Figure S5). Stronger effects have been observed when *asm-3* is co-inactivated with *asm-1* or *asm-2* (Figure 4B; Figure S6). Nuclear translocation of DAF-16::GFP fusion protein has also been observed when *daf-2*, *age-1* or *akt-1* is inactivated by RNAi (Figure 4A; Figure S5). Consistently, qRT-PCR analysis has shown that several known DAF-16 downstream target genes, including *sod-3,* are also up-regulated when *asm-3* or *daf-2* is inactivated by mutations (Figure 5A). Together, our data indicate that *asm-3* gene activity regulates the sub-cellular localization and transcriptional activity of DAF-16/FOXO in a manner similar to *daf-2*.

In addition, we have found that DAF-16 protein levels are up-regulated not only in the *asm-3(ok1744)* mutants and in animals treated with *asm-3(RNAi)*, but also in the *daf-2(e1370)* mutants or *daf-2(RNAi)* animals (Figure 4C, 4D). It is possible that an additional role of DAF-2 in regulation of DAF-16 is mediated through the regulation of DAF-16 protein levels, and that this process also involves ASM-3. Up-regulation of DAF-16 protein levels has been previously observed in *rle-1* mutants and *eak-7* mutants, both known to have a longer animal lifespan [12], [40]. Future studies are needed to determine how DAF-2 and ASM-3 regulate DAF-16 protein levels.

### A Potential Mechanism of ASM-3 in the Regulation of Trans-membrane Receptor Signaling

The predicted *C. elegans* ASM-3 peptide shares a strong homology with the human ASM in the presumed catalytic domain of the ASM enzyme (Figure 1S). In cultured human cells, ASM-dependant ceramide production in lipid rafts has been shown to be essential for CD95 death receptor mediated signaling to apoptosis [18], [19], [53]. However, the endogenous CD95 receptors have recently been found to have a pro-survival and pro-proliferation function in the mouse knockout system [20], [21]. It appears that the biological consequence of CD95 receptor signaling is dependent on cell context and stimulus used. It remains to be investigated whether the pro-proliferation and pro-survival signaling by the CD95 receptors requires the participation of ASM.

Our studies have provided strong genetic evidence that ASM-3 is a positive regulator of signaling through the DAF-2 receptors. This is the first report that ASM functions *in vivo* as a positive regulator for a receptor tyrosine kinase. By analogy with human ASM in the formation of ceramide-enriched lipid rafts, it is possible that similar lipid rafts may be involved in facilitating the DAF-2 receptor signaling in *C. elegans*. In light of the fact that the strongest phenotypes of *asm-3* inactivation are observed in *age-1* mutant backgrounds, it can be further speculated that transducing the signals from DAF-2 receptor to the AGE-1 PI 3-kinase homolog may involve ASM-3-dependent lipid rafts. In support of this hypothesis, we have observed that, in mammalian systems, human ASM regulates the lipid raft localization of the IGF-1 receptors (P. Ghosh, Y. Kim, X. Xiong and H. Sun, unpublished observations). Taken together, our studies indicate that ASM-3 proteins, along with their products ceramides, play a positive role in facilitating DAF-2 receptor signaling.

### *asm-3* Serves as a Molecular Target for Anti-aging

Our genetic studies have shown that *asm-3* regulates animal lifespan through modulation of the *daf-2*/IIS pathway. These results suggest the potential of using ASM as a molecular target for anti-aging at organismal level. Indeed, we have shown that two chemical compounds, desipramine and clomipramine, which are known to inhibit mammalian ASM activity [50]–[52], can effectively extend wild-type animal lifespan by 24% or 14%, respectively (Figure 6A, 6B). Such lifespan extension phenotypes require the presence of *daf-16*, as no lifespan extension was observed in the *daf-16(mgDf47)* null mutant background (Figure 6C, 6D). The effects of these compounds on lifespan are very similar to the lifespan extension resulting from genetic inactivation of the *asm* gene family (Figure 1). Consistently, our biochemical studies have demonstrated that these compounds indeed greatly decrease the CeASM activities (Figure 6E). Thus, these data suggest that these two drugs mediate their effects through inhibition of CeASMs. Together, these results further underscore the importance of ASM in the regulation of animal lifespan and its role as a potential target for anti-aging. Moreover, since mammalian ASM is known to localize to the outer leaflet of the plasma membrane [16], ASM presents itself as an even more attractive target for anti-aging drug development.

## Materials and Methods

### C. elegans Strains

We used N2 Bristol as the wild-type strain. In addition, strains carrying the following mutant alleles were used: *asm-3(ok1744)*, *daf-2(e1370)*, *age-1(mg305)*, *aap-1(m889)*, *pdk-1(sa709)*, *akt-1(mg306)*, *daf-16(mg26)*, *daf-16(mgDf47)*, *daf-18(nr2037)*, *daf-7(e1372)*, *rrf-3(pk1426)*, *asm-3(ok1744);daf-2(e1370)*, *asm-3(ok1744);age-1(mg305)*, *asm-3(ok1744);pdk-1(sa709)*, *asm-3(ok1744);akt-1(mg306)*, *asm-3(ok1744);daf-16(mgDf47)*, *asm-3(ok1744);daf-7(e1372)*, *asm-3(ok1744);rrf-3(pk1426)*, *daf-18(nr2037);rrf-3(pk1426)*, *rrf-3(pk1426);zIs356[daf-16::daf-16::gfp rol-6(su1006)*], *asm-3(ok1744)*;*rrf-3(pk1426);zIs356[daf-16::daf-16::gfp rol-6(su1006)*], *rrf-3(pk1426);muIs84[pAD76(sod-3::gfp*)], *asm-3(ok1744);rrf-3(pk1426);muIs84 [pAD76(sod-3::gfp*)]. The *asm-3(ok1744)* strain was obtained from *C. elegans* Genetic Center (CGC), and backcrossed to the wild-type N2 strain for 3 times. The *daf-18(nr2037)* strain was described previously [15]. All other strains were also obtained from CGC except *age-1(mg305)*, *akt-1(mg306)* and *pdk-1(sa709)* which are kindly provided by Dr. Patrick Hu (University of Michigan, Ann Arbor). All the double mutant strains were constructed by standard genetic techniques and the presence of homozygous mutant alleles were confirmed by PCR genotyping, restriction fragment length polymorphisms or strong dauer phenotype at 25°C or 27°C. All strains used in this study were maintained at 15°C using standard techniques for the control of *C. elegans* [55]. *E. coli* OP50 was used as the food source on NGM plates.

### Construction of *asm-1* and *asm-2* RNAi Clones

The constructs were based on PCR strategy from Worm Base. *asm-1* and *asm-2* amplicons, sjj_B0252.2(1141bp) and sjj_ZK455.4(1107bp), respectively, were amplified from genomic DNA of N2 wild-type worms by PCR and subcloned to TOPO TA cloning vector (pCR2.1-TOPO, Invitrogen). The following primers were used for *asm-1* RNAi construct: Forward: 5′-tcagtcgacgaacgattctg-3′; Reverse: 5′-ctctcgtttcttttcgctgg-3′. The primers used for *asm-2* RNAi construct are as follows: Forward: 5′-tggaatccaatgagaccaca-3′; Reverse: 5′-tgctagtcaatttcccgctt-3′. Unique restriction sites from the subcloned vector were used for cloning into the L4440 vector (a kind gift from Dr. A. Fire). The derived RNAi constructs were confirmed by restriction mapping and DNA sequencing. The constructs were transformed into *E. coli* HT115 (DE3) strain [56]. The *asm-3*, *daf-16* and *daf-18* RNAi constructs were recovered from the RNAi feeding library [57], [58] and confirmed by DNA sequencing. The *daf-2* RNAi construct has been described previously [25].

### Lifespan Assay on NGM Agar Plates

Lifespan assays were carried out on NGM plates at 20°C, and plates were seeded with *E. coli* OP50. In order to synchronize worms, eggs were first isolated from gravid adults and then hatched overnight in the S1 basal media. The synchronized L1 larva were then placed on OP50-seeded NGM agar plates and allowed to grow to adult. During the egg-laying period, adult worms were daily transferred to new OP50-seeded agar plates. Worms were then examined every other day for survival and scored. Where indicated, FuDR was also included in the plates. FuDR, 5-Fluoro-2′-deoxyuridine, is used to inhibit DNA synthesis and thus to prevent the production of progeny. Animals hatched from egg preparation were grown on regular OP50-seeded NGM agar plates until L4 stage and then transferred to plates containing 50 µg/ml FuDR. Animal survival was scored every other day and transferred to new plates containing 50 µg/ml FuDR every 2∼3 days. Animals were considered dead when failed to respond to gentle touches by a platinum wire. Adult lifespan were counted using the L4 stage as Day 0. For each set of experiments, animals from different strains or conditions were assayed in parallel. Each set of experiments was carried out at least 2 times. In all the lifespan assays performed on regular NGM plates, RNAi-inducing plates or drug-containing plates, at least 50 worms were counted per strain per experiment; while the animals that were bagged, exploded or crawled off the plate were censored from the counts.

### Lifespan Assay on RNAi-inducing Plates

RNAi bacteria culture was grown in Luria broth media with 100 µg/ml ampicillin at 37°C for 16∼18 hours and then seeded onto NGM plates containing 5 mM IPTG and 50 µg/ml carbanicillin. In case of double knockdown, RNAi bacteria cultures of same density were mixed at 1∶1 ratio and were then seeded on RNAi-inducing plates. The plates were kept at room temperature overnight to induce the expression of the RNAi molecules. Lifespan assay was previously described [25]. Briefly, the synchronized L1 larvae animals prepared by egg preparation were plated on the RNAi-inducing plates and allowed to grow to adults. The adult animals were transferred to new plates every day until adult animals stop laying eggs and after that the animals were scored every other day and transferred to new plates every 2∼3 days. All the RNAi experiments were carried out in the *rrf-3(pk1426)* background, a strain known to be hypersensitive to RNAi, with the exception of RNAi experiment in the *aap-1(m889)* background carrying the *rrf-3(+)* allele. Lifespan assays were carried out at 20°C. Each set of experiments were conducted at least 2 times.

### Lifespan Assay on Drug-containing NGM Plates

To prepare NGM plates containing desipramine or clomipramine, drugs were first dissolved in water as 100 mM stock solutions and sterilized by filtration. Drugs were then properly diluted to the indicated final concentration during the solidification step of the NGM agar plate preparation. The synchronized L1 larvae prepared by egg preparation were placed on NGM agar plates and allowed to develop to L4 larva. Synchronized L4 animals were then transferred to regular NGM (control) plates or plates containing drug desipramine (30 µM) or clomipramine (5 µM). Animals were then transferred to drug-containing plates or regular NGM plates every day until animals stop laying eggs. The populations were scored every other day and transferred to new drug-containing plates or regular NGM plates every 2∼3 days. Adult lifespan was counted using the L4 stage as Day 0. Lifespan assays were carried out at 20°C. Drug inhibition experiments have been carried out >3 times and consistent results have been obtained from independent experiments.

### Statistical Analysis

Statistical analyses of all the survival curves were performed as described [11] using the software GraphPad 5.0. Survival curve of each population was compared with control using Log-rank (Mantel-Cox) test and an experimental data set with a P value <0.05 was considered to be significantly different from the control data set.

### Oxidative Stress Response Assay using Paraquat

The synchronized L1 animals after standard egg preparation were placed on NGM agar plates seeded with *E. coli* OP50 and allowed to develop to the L4 stage larva at 20°C. A 1-mL 80 mM paraquat solution was then treated on the 6-cm plates at the L4 stage and after soaking for about 20 minutes, the plates were air-dried in a chemical hood till plates were dried. The dried plates were shifted to 20°C incubator and survivor of the animals was scored every day. The experiment was repeated at least two independent times. Total worms counted per plate were greater than 100.

### Heat Stress Response Assay at 35°C

The synchronized L1 animals after egg preparation were put on NGM agar plates seeded with *E. coli* OP50 and allowed to grow to Day 1 stage worms (young adult worms) at 20°C. At Day 1, the plates were shifted to 35°C and then scored after 6, 9, 12 or 15 hours afterwards. The plates used for counting in each time point were discarded to avoid the complication of recovery at room temperature on scoring plates. Experiments were conducted for two times, each in triplicates, and the standard deviations were calculated. The number of worms counted per strain per plate was greater than 200.

### Dauer Assay

All the strains used for dauer assay were synchronized by egg preparation and the synchronized L1 larvae were placed on NGM agar plates seeded with *E. coli* OP50. The plates were incubated at 22.5°C, 25°C or 27°C. Numbers of dauer and nondauer in the population were visually scored after 72, 48 and 45 hours later for the assays carried out at 22.5°C, 25°C or 27°C, respectively. All assays were performed in triplicates and experiments were conducted at least 2 times.

### DAF-16::GFP Localization and *sod-3p*::GFP Expression Assay

We crossed the *rrf-3(pk1426)* or the *asm-3(ok1744);rrf-3(pk1426)* allele into wild-type strain carrying the *daf-16::gfp* or *sod-3p::gfp* transgene and derived the following GFP-expressing transgenic strains : 1) *rrf-3(pk1426); zIs356[daf-16::daf-16::gfp, rol-6(su1006)]*; 2) *rrf-3(pk1426);asm-3(ok1744)*; *zIs356[daf-16::daf-16::gfp, rol-6(su1006)]*; 3) *rrf-3(pk1426);muls84[pAD76(sod-3::gfp)]*; 4) *asm-3(ok1744);rrf-3(pk1426);muIs84 [pAD76(sod-3::gfp)]*. The GFP-expressing transgenic strains were synchronized at L1 larvae stage in the S1 basal media after standard egg preparation and placed onto RNAi-inducing bacteria plates at 20°C. All the worms were transferred to new RNAi-inducing bacteria plates every day thereafter. For fluorescence microscopy, worms were transferred to 2% agarose pads and examined under Olympus CKX41 fluorescence microscope with 4x objective. Worms were photographed with the attached digital camera QICAM FAST1394. For image comparison of individual conditions in each set of experiments, identical exposure times were used for image capture. Animals were examined on adult Day 1, Day 3 or Day 4 as described in the Figure legends, with the L4 stage counted as adult Day 0.

### RNA Isolation and qRT-PCR

Total RNA was isolated from synchronized adult Day 1 worms using Trizol reagent (Invitrogen). RNA purity was checked by UV absorbance (260/280 ratio). cDNA was synthesized with primer Oligo(dT)_20_ by using SuperScript III First-Strand Kit (Invitrogen) according to the manufacturer’s protocol. RT-PCR reactions were performed with a 20 µl of Power SYBR PCR Master Mix (Applied Biosystems) using triplicates for each sample. PCR reaction was carried out on Real Time PCR Machine 7500 Fast (Applied Biosystems) under the condition of 95°C for 5 minutes for denaturation, followed by 40 cycles of 95°C for 15 seconds and 60°C for 1 hour. The reaction products were analyzed using software provided by the onboard software from the Real Time PCR machine. The primers used for qRT-PCR are as follows: Primer for *act-1* (133 bp in product size) [34], [39]; Forward primer: 5′-ccaggaattgctgatcgtatgcagaa-3′; Reverse primer: 5′-tggagagggaagcgaggataga-3′: Primer for *sod-3* (98 bp in product size) [34], [39]; Forward primer: 5′-tcgcactgcttcaaagcttgttcaa-3′; Reverse primer: 5′-ccaaatctgcatagtcgaatgggagat-3′: Primer for *mtl-1* (111 bp in product size); Forward primer: 5′-atggcttgcaagtgtgactg-3′; Reverse primer: 5′-tttctcactggcctcctcac-3′. *act-1* was used as an internal control for normalization of input RNA levels. All experiments have been repeated >2 times and consistent results have been obtained from independent experiments.

### Preparation of Worm lysate and Western Blot Analysis

Synchronized young adult worms (at adult Day 1) were rinsed three times with M9 buffer to remove bacteria and then washed one time with ddH_2_O to remove salt. 0.4 volume of 5X SDS-PAGE sample buffer (312.5 mM Tris-HCl, pH 6.8, 50% Glycerol and 10% SDS) was added to each tube containing the packed worms. Tubes were subjected to 3 cycles of freezing/heating (CO_2_/ethanol bath and boiling at 95°C for 10 minutes). Samples were then sonicated using water bath sonicator (VWR B1500A-MT) for 3 cycles of 2 minutes per cycle. Lysates were clarified by centrifugation at 12000 rpm for 1 minute and supernatants were transferred to new tubes, and protein concentrations were quantified using BCA protein assay reagent (Thermo Scientific). For experiments involving RNAi-treated samples, adult Day 2 worms from plates seeded with RNAi-inducing bacteria were used. Equal amounts of protein lysates were analyzed by SDS-PAGE analysis, and the proteins were transferred to nitrocellulose membrane. Membranes were blocked with 1X TBST (150 mM NaCl, 20 mM Tris-base, pH 7.4 and 0.05% Tween-20) containing 5% non-fat dry milk for 1 hour at room temperature. Primary antibody were applied and incubated at 4°C overnight. Filters were extensively washed, and then incubated with a properly diluted secondary antibody for 1 hour at room temperature. Following washes, filters were developed with Western lightning Plus-ECL (Perkin Elmer) and exposed to X-ray films. The primary antibodies used were antibodies specific for DAF-16 (1∶400 dilution) (cN-20, Santa Cruz Biotechnology, INC) and β-ACTIN (1∶2000 dilution) (Cell Signaling Technology). Secondary antibodies used were Donkey anti-goat IgG-HRP (1∶2000 dilution) (Santa Cruz Biotechnology, INC) and Peroxidase-conjugated IgG fraction monoclonal Mouse anti-rabbit IgG, light chain specific (1∶2000 dilution) (Jackson ImmunoResearch Laboratories). Membranes were first probed with anti-DAF-16 antibody and filters were then stripped by incubation at 56°C for 30 minutes in the stripping buffer (2% SDS, 62.5 mM Tris-HCl pH6.8 and 100 mM of β-mercaptoethanol). Filters were then extensively washed with 1X TBST solution and then reprobed with anti-β-actin antibody. The amount of β-actin in each sample serves as an internal loading control.

### Acid Sphingomyelinase Assay

Synchronized wild type L1 larvae animals prepared by egg preparation were placed on regular NGM plates (control) or plates containing drug desipramine (30 µM) or clomipramine (5 µM) and allowed to develop to L3 larva for 30 hours. At L3 stage, worms were further treated with either ddH_2_O, desipramine (30 µM) or clomipramine (20 µM) by adding 500 µl of the corresponding solution to each plate. The plates were dried for 20 minutes in air-ventilated hood and then returned to the 20°C incubator for 18 hours. At L4 stage, worms were harvested using M9 buffer. Worms were washed three times with M9 buffer followed by two times washes with ddH_2_O. To each tube of packed worm (∼800 µl), 500 µl of cold lysis buffer (0.2% Triton X-100, 50 mM Tris-HCl pH 7.4, 50 mM sodium acetate, 1 mM EDTA and protease inhibitors) was added and samples were incubated on ice for 30 minutes. Samples were then sonicated using a water bath sonicator (Branson 1510) for 20 seconds then returned to ice for 1 minute and the procedure was repeated 5 times. Lysates were clarified by centrifugation at 800×*g* for 5 minutes at 4°C. The clarified lysates were measured for protein concentration using Bio-Rad protein assay kit and then normalized. CeASM activity assays were measured according to the procedures described by (24). [^14^C]-sphingomyelin (Amersham Life Sciences, supplied in organic solvent) was air-dried then reconstituted in the assay buffer (250 mM sodium acetate pH5.0, 1 mM EDTA and 0.1% Triton X-100) and briefly sonicated in water bath sonicator. The assay was initiated by combining 200 µl of lysate with 200 µl assay buffer containing [^14^C]-sphingomyelin (25 nCi/sample) and then incubated at 37°C for 40 minutes. Reaction mixtures were subsequently extracted with 800 µl chloroform/methanol mixture (2∶1 v/v). After centrifugation at 5000×*g* for 5 minutes, an aliquot of the aqueous phase (600 µl) were removed and counted by liquid scintillation counter (Beckman Coulter LS 6500). All samples were assayed in duplicate, and the lysis buffer alones were served as negative controls.

### Quantification of DAF-16 Protein Levels

The relative abundance of DAF-16 protein levels were calculated from the western blots using the ImageJ software. The intensity of DAF-16 was normalized against beta-actin, and the numbers were presented as a percentage relative to the N2 or the vector control. The controls were set as 100%.

## Supporting Information

Figure S1 **Multiple alignment of** ***C. elegans*** **ASM-3,** ***H. sapiens*** **ASM and** ***D. melanogaster*** **ASM.** The C-terminal catalytic domain of CeASM-3 was aligned with that of HsASM and DmASM. Sequences used in the alignment correspond to amino acids 117 to 464 of CeASM-3, 186 to 523 of HsASM and 254 to 596 of DmASM. Identical residues are shaded black and similar residues are shaded gray. CeASM-3 shares 42% identity with HsASM and 39% identity with DmASM. Alignment was performed using Clustal W2 and BioEdit v7.0.5. Consensus symbol “*” is used when the residues in all sequences are identical. Symbol “:” or “·” is used for conserved substitutions or semi-conserved substitutions, respectively.
(TIF)

Figure S2 **Genomic structures of** ***asm-3*** **and** ***ok1744*** **allele.** The genomic arrangement of *asm-3* is illustrated according to the Worm database (www.wormbase.org). The *ok1744* allele contains a deletion of 1558 bp which removes exon VII through exon XIII of *asm-3*, followed by a 7 bp insertion. As a consequence, such changes shall lead to production of a peptide that is truncated at the amino acid 272 (Pro272).
(TIF)

Figure S3 **Multiple alignment of** ***C. elegans*** **ASM-3, ASM-1 and ASM-2.** The predicted C-terminal catalytic domain of CeASM-3 was aligned with that of CeASM-1 and CeASM-2. Sequences used in the alignment correspond to amino acids 117 to 464 of CeASM-3, 138 to 478 of CeASM-1 and 167 to 526 of CeASM-2. Identical or similar residues are shaded black or gray, respectively. CeASM-3 shares 39% identity with CeASM-1 and 41% identity with CeASM-2. Alignment was performed using Clustal W2 and BioEdit v7.0.5. Consensus symbol “*” is used when the residues in all sequences are identical. Symbol “:” or “·” is used for conserved substitutions or semi-conserved substitutions, respectively.
(TIF)

Figure S4 **Stress responses.** (A) Loss of *asm-3* resulted in an increase in resistance towards oxidative stress using paraquat treatment (80 mM). (B) Loss of *asm-3* promoted resistance towards heat stress (35°C). In (A) and (B), *daf-16(mgDf47)* mutants were sensitive to oxidative stress or heat stress. Increased resistance of the *asm-3(ok1744)* mutants was dependent on the *daf-16* gene activity. For all stress response assays, experiments were repeated at least two independent times with similar results obtained. Data from representative sets of experiments are shown. For the oxidative stress assays, greater than 100 worms were counted for each strain in each experiment. For the heat stress assays, greater 200 worms per plate per strain were counted and experiments were conducted in triplicates. Error bars indicate standard deviation.
(TIF)

Figure S5 **Examination of DAF-16::GFP subcellular localization.** The subcellular localization of DAF-16::GFP fusion protein was examined by fluorescence microscopy. *rrf-3(pk1426);daf-16::gfp* mutants were treated with the indicated RNAi molecules and examined on adult day 4. For the *daf-2* and *akt-1* RNAi, animals were examined on adult day 1 and day 4. In the vector alone (L4440) control animals, DAF-16::GFP proteins were localized only in the cytoplasm. In animals treated with RNAi molecules for *asm-3*, *asm-1* or *asm-2*, DAF-16::GFP proteins were localized mostly in the nucleus. As positive controls, RNAi of *daf-2*, *age-1* or *akt-1* each induced the nuclear localization of DAF-16::GFP proteins. RNAi knockdown of *daf-16*, a negative control, markedly reduced DAF-16::GFP expression. In *daf-18* RNAi-treated animals, no nuclear translocation of DAF-16::GFP was observed.
(TIF)

Figure S6 **Synergistic effect of DAF-16::GFP nuclear localization.** DAF-16::GFP cellular distributions were examined by fluorescence microscopy. *asm-3(ok1744);rrf-3(pk1426);daf-16::gfp* mutant animals were examined on adult day 4. In the vector alone treated animals, DAF-16::GFP proteins were partially nuclear-localized due to the presence of *asm-3(ok1744)* allele. Further inactivation of *asm-1*, *asm-2*, or *asm-1* together with *asm-2* (*asm-1/asm-2*) by RNAi induced more pronounced nuclear localization of DAF-16::GFP. RNAi inactivation of *daf-16*, a negative control, markedly reduced DAF-16::GFP expression. The *daf-18* RNAi prevented the nuclear localization of DAF-16::GFP proteins caused by loss of *asm-3.*
(TIF)

Table S1 **Effects of** ***asm-3*** **on dauer formation.** Dauer formation experiments were carried out for the wild-type animals and various mutants at the indicated temperatures. Dauer formation assays were conducted in triplicates and experiments were repeated at least two times. Data from representative experiments are shown. The average percentages of nondauer and dauer, as well total numbers of animals used for each assay, were listed.
(PDF)
