# Supplementary figures and images for: ASM-3 Acid Sphingomyelinase Functions as a Positive Regulator of the DAF-2/AGE-1 Signaling Pathway and Serves as a Novel Anti-Aging Target
Source: PLoS One. 2012 Sep 25;7(9):e45890. doi: 10.1371/journal.pone.0045890 (PMC3457945; doi:10.1371/journal.pone.0045890)

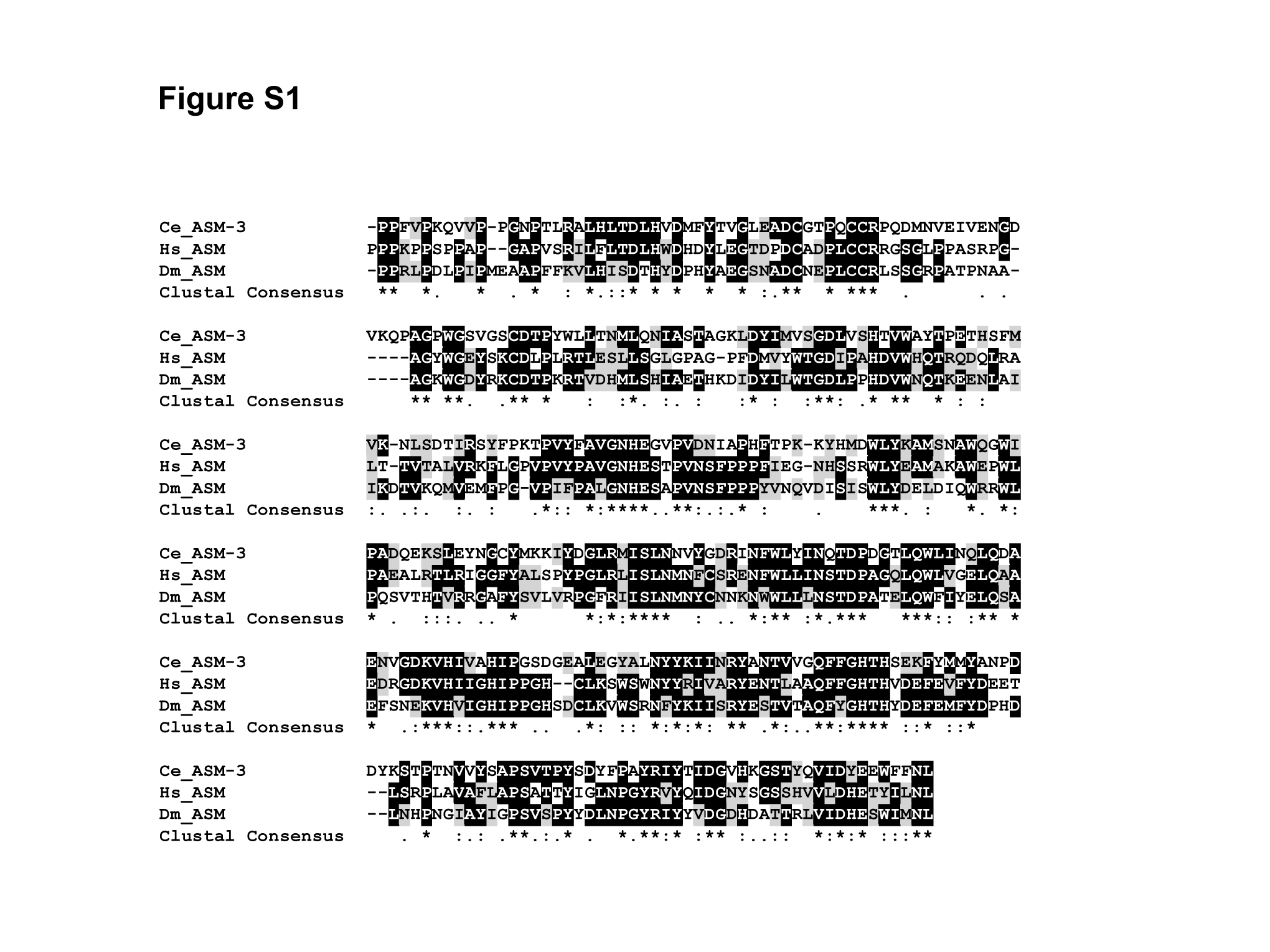

Supplement: Figure S1 — Multiple alignment of C. elegans ASM-3, H. sapiens ASM and D. melanogaster ASM. The C-terminal catalytic domain of CeASM-3 was aligned with that of HsASM and DmASM. Sequences used in the alignment correspond to amino acids 117 to 464 of CeASM-3, 186 to 523 of HsASM and 254 to 596 of DmASM. Identical residues are shaded black and similar residues are shaded gray. CeASM-3 shares 42% identity with HsASM and 39% identity with DmASM. Alignment was performed using Clustal W2 and BioEdit v7.0.5. Consensus symbol “*” is used when the residues in all sequences are identical. Symbol “:” or “·” is used for conserved substitutions or semi-conserved substitutions, respectively. (TIF) [file pone.0045890.s001.tif]

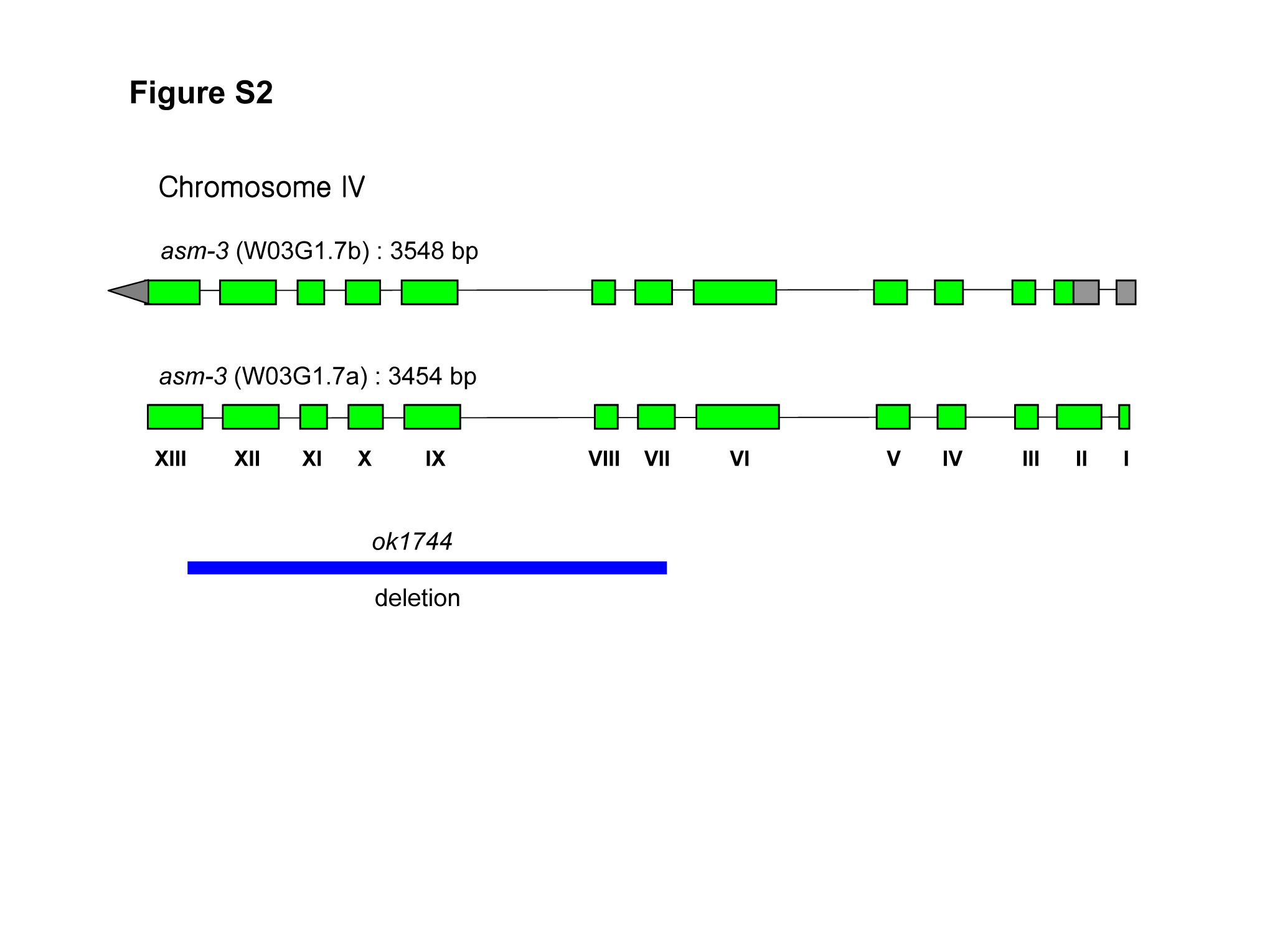

Supplement: Figure S2 — Genomic structures of asm-3 and ok1744 allele. The genomic arrangement of asm-3 is illustrated according to the Worm database (www.wormbase.org). The ok1744 allele contains a deletion of 1558 bp which removes exon VII through exon XIII of asm-3, followed by a 7 bp insertion. As a consequence, such changes shall lead to production of a peptide that is truncated at the amino acid 272 (Pro272). (TIF) [file pone.0045890.s002.tif]

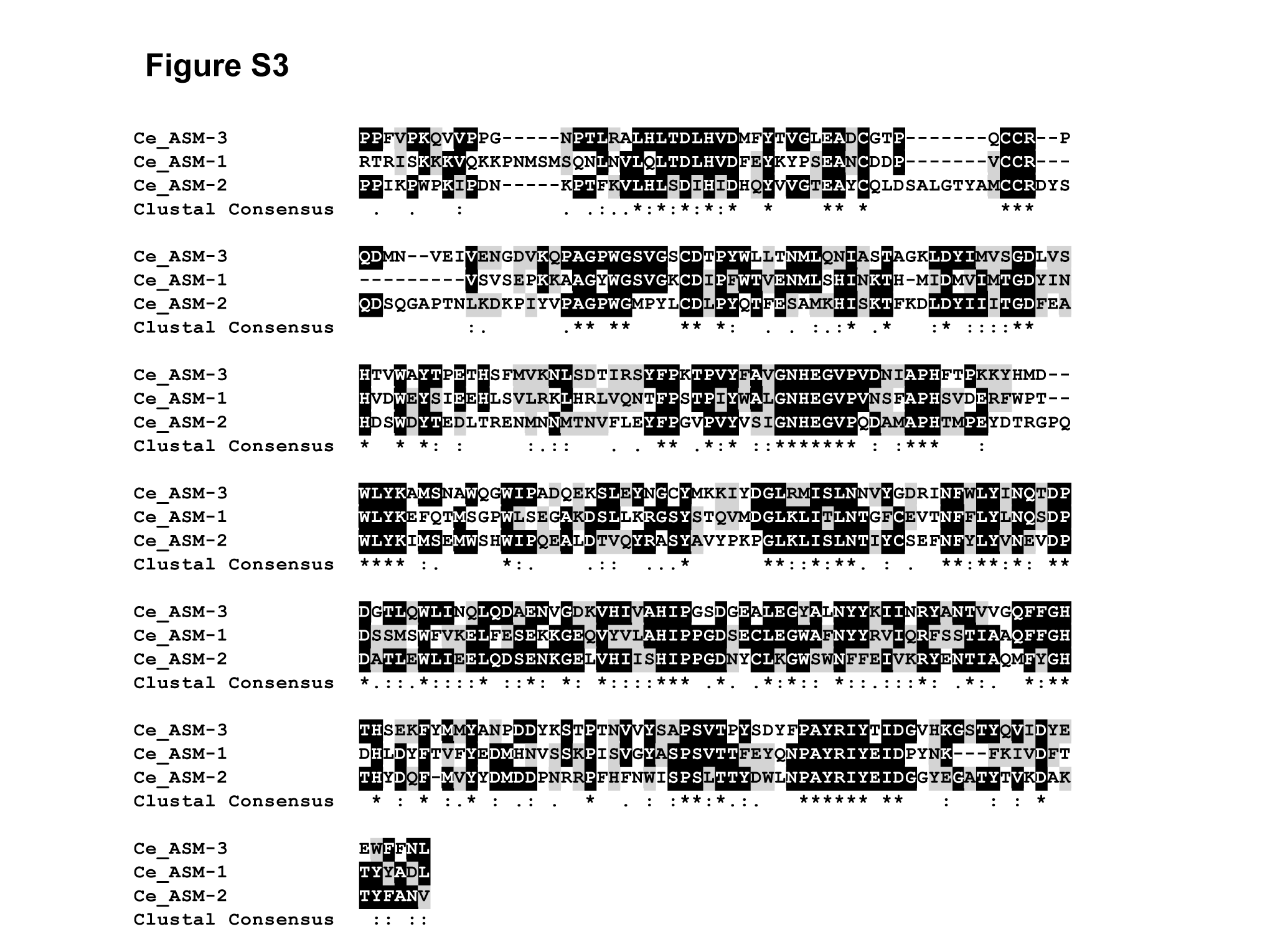

Supplement: Figure S3 — Multiple alignment of C. elegans ASM-3, ASM-1 and ASM-2. The predicted C-terminal catalytic domain of CeASM-3 was aligned with that of CeASM-1 and CeASM-2. Sequences used in the alignment correspond to amino acids 117 to 464 of CeASM-3, 138 to 478 of CeASM-1 and 167 to 526 of CeASM-2. Identical or similar residues are shaded black or gray, respectively. CeASM-3 shares 39% identity with CeASM-1 and 41% identity with CeASM-2. Alignment was performed using Clustal W2 and BioEdit v7.0.5. Consensus symbol “*” is used when the residues in all sequences are identical. Symbol “:” or “·” is used for conserved substitutions or semi-conserved substitutions, respectively. (TIF) [file pone.0045890.s003.tif]

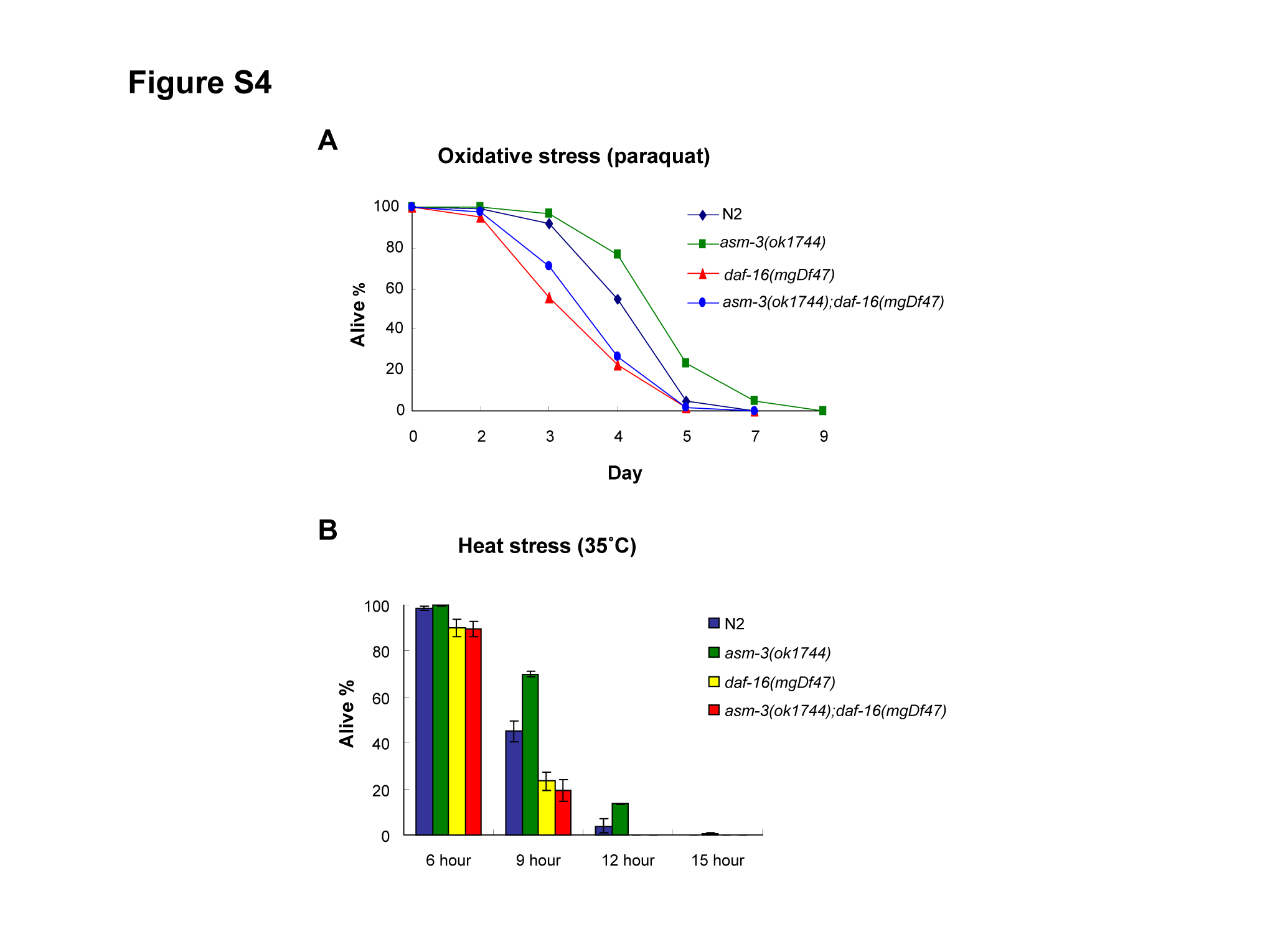

Supplement: Figure S4 — Stress responses. (A) Loss of asm-3 resulted in an increase in resistance towards oxidative stress using paraquat treatment (80 mM). (B) Loss of asm-3 promoted resistance towards heat stress (35°C). In (A) and (B), daf-16(mgDf47) mutants were sensitive to oxidative stress or heat stress. Increased resistance of the asm-3(ok1744) mutants was dependent on the daf-16 gene activity. For all stress response assays, experiments were repeated at least two independent times with similar results obtained. Data from representative sets of experiments are shown. For the oxidative stress assays, greater than 100 worms were counted for each strain in each experiment. For the heat stress assays, greater 200 worms per plate per strain were counted and experiments were conducted in triplicates. Error bars indicate standard deviation. (TIF) [file pone.0045890.s004.tif]

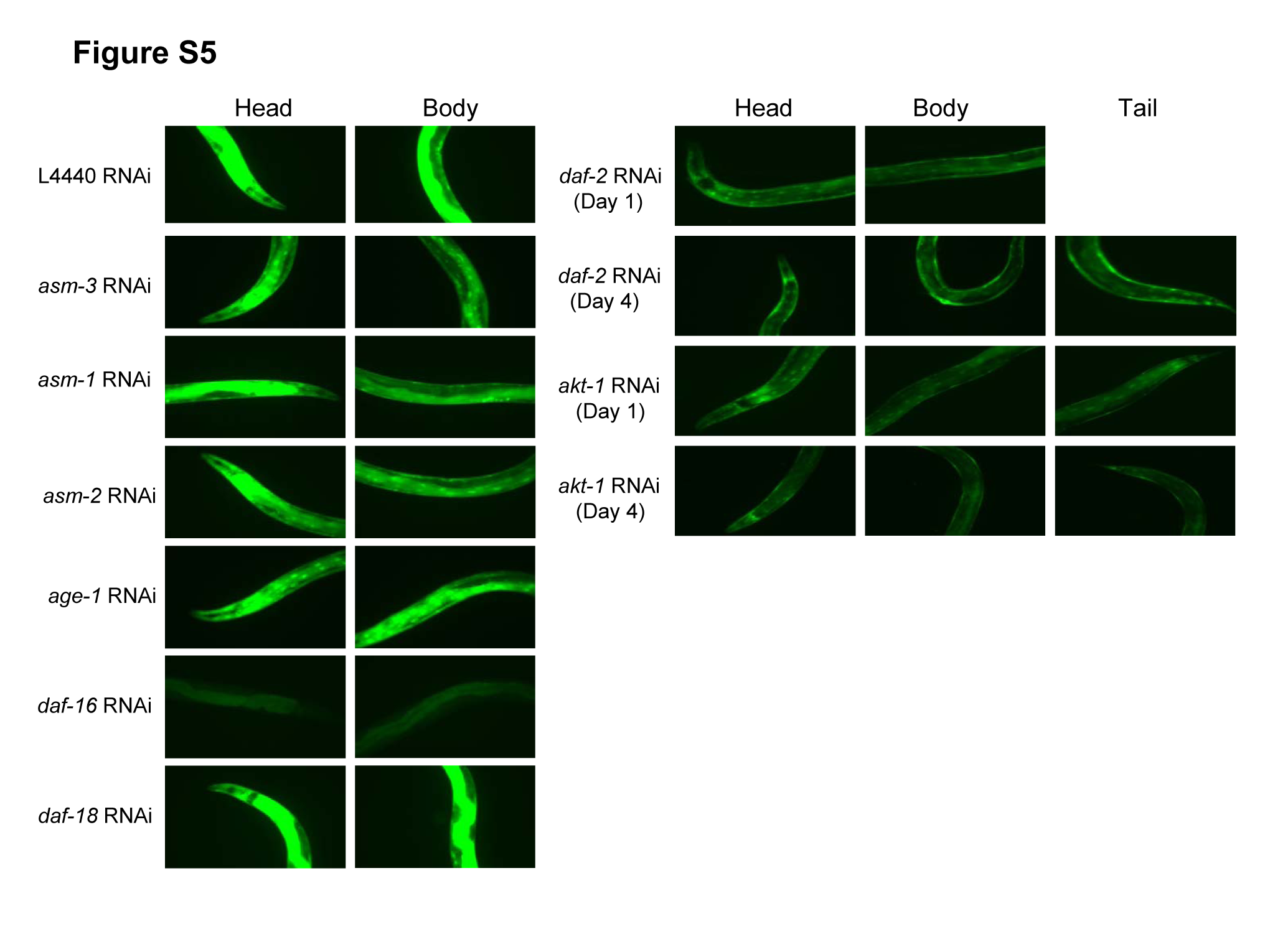

Supplement: Figure S5 — Examination of DAF-16::GFP subcellular localization. The subcellular localization of DAF-16::GFP fusion protein was examined by fluorescence microscopy. rrf-3(pk1426);daf-16::gfp mutants were treated with the indicated RNAi molecules and examined on adult day 4. For the daf-2 and akt-1 RNAi, animals were examined on adult day 1 and day 4. In the vector alone (L4440) control animals, DAF-16::GFP proteins were localized only in the cytoplasm. In animals treated with RNAi molecules for asm-3, asm-1 or asm-2, DAF-16::GFP proteins were localized mostly in the nucleus. As positive controls, RNAi of daf-2, age-1 or akt-1 each induced the nuclear localization of DAF-16::GFP proteins. RNAi knockdown of daf-16, a negative control, markedly reduced DAF-16::GFP expression. In daf-18 RNAi-treated animals, no nuclear translocation of DAF-16::GFP was observed. (TIF) [file pone.0045890.s005.tif]

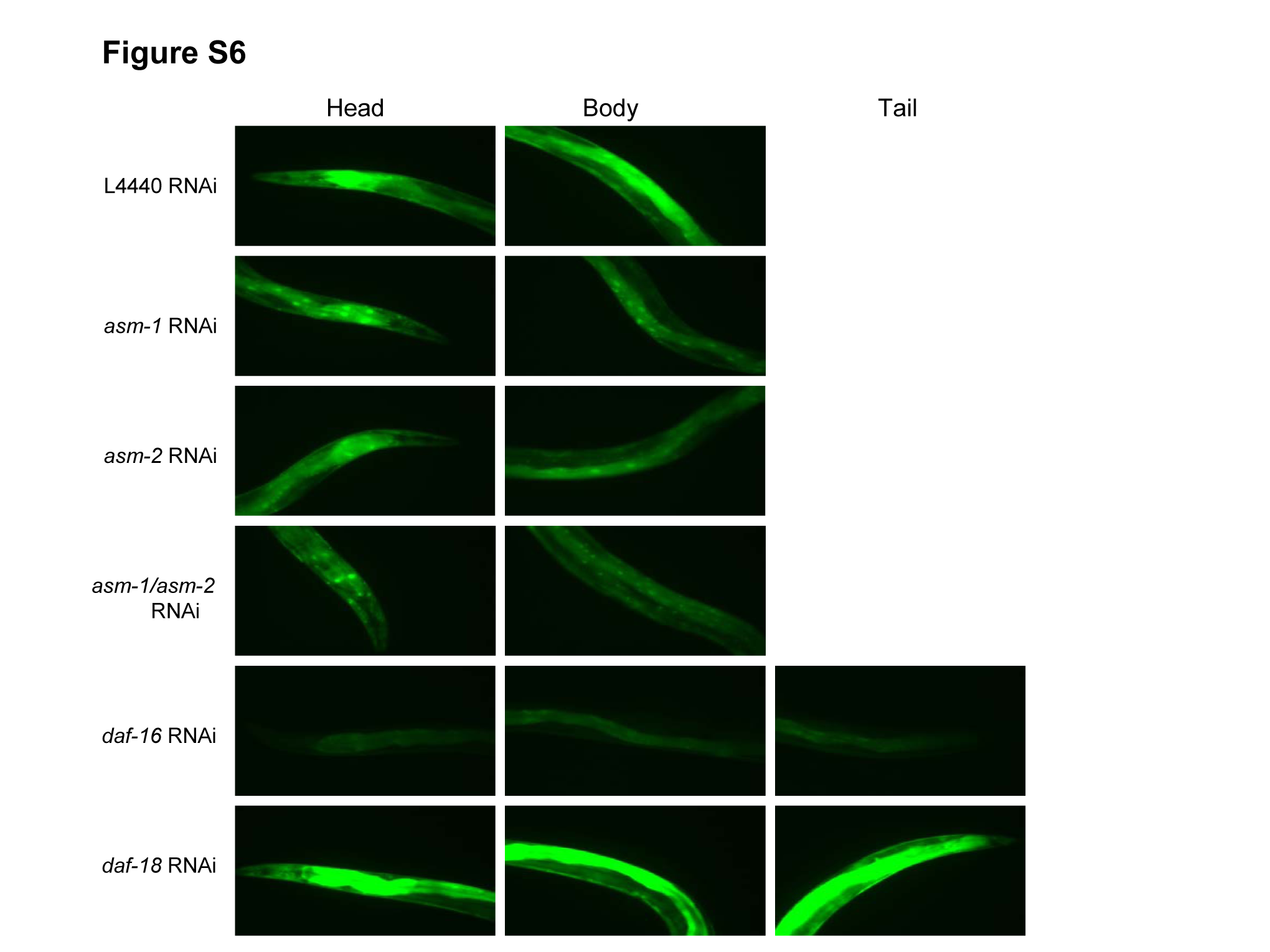

Supplement: Figure S6 — Synergistic effect of DAF-16::GFP nuclear localization. DAF-16::GFP cellular distributions were examined by fluorescence microscopy. asm-3(ok1744);rrf-3(pk1426);daf-16::gfp mutant animals were examined on adult day 4. In the vector alone treated animals, DAF-16::GFP proteins were partially nuclear-localized due to the presence of asm-3(ok1744) allele. Further inactivation of asm-1, asm-2, or asm-1 together with asm-2 (asm-1/asm-2) by RNAi induced more pronounced nuclear localization of DAF-16::GFP. RNAi inactivation of daf-16, a negative control, markedly reduced DAF-16::GFP expression. The daf-18 RNAi prevented the nuclear localization of DAF-16::GFP proteins caused by loss of asm-3. (TIF) [file pone.0045890.s006.tif]
