# Supplementary material for: ASM-3 Acid Sphingomyelinase Functions as a Positive Regulator of the DAF-2/AGE-1 Signaling Pathway and Serves as a Novel Anti-Aging Target
Source: PLoS One. 2012 Sep 25;7(9):e45890. doi: 10.1371/journal.pone.0045890 (PMC3457945; doi:10.1371/journal.pone.0045890)
Supplement: Table S1 — Effects of asm-3 on dauer formation. Dauer formation experiments were carried out for the wild-type animals and various mutants at the indicated temperatures. Dauer formation assays were conducted in triplicates and experiments were repeated at least two times. Data from representative experiments are shown. The average percentages of nondauer and dauer, as well total numbers of animals used for each assay, were listed. (PDF) [file pone.0045890.s007.pdf]

Table S1

Strain	nondauer (%)	dauer (%)	Total Number	nondauer (%)	dauer (%)	Total Number
		22.5˚C			25˚C	
wild-type (N2)	100	0	279	100	0	810
asm-3(ok1744)	100	0	246	100	0	846
daf-2(e1370)	53.6	46.4	162	0	100	798
asm-3(ok1744);daf-2(e1370)	14	86	157	0	100	873
		22.5°C			25°C	
wild-type (N2)	100	0	562	100	0	752
asm-3(ok1744)	100	0	617	100	0	765
age-1(mg305)	97	3	687	0.9	99.1	853
asm-3(ok1744);age-1(mg305)	0.9	99.1	611	0.5	99.5	719
		25°C			27°C	
wild-type (N2)	100	0	712	96	4	658
asm-3(ok1744)	100	0	709	95.5	4.5	710
pdk-1(sa709)	100	0	801	0.4	99.6	631
asm-3(ok1744);pdk-1(sa709)	100	0	750	0.6	99.4	688
		25˚C			27°C	
wild-type (N2)	100	0	373	100	0	518
asm-3(ok1744)	100	0	430	100	0	504
akt-1(mg306)	100	0	259	7.2	92.8	672
asm-3(ok1744);akt-1(mg306)	100	0	799	37.2	62.8	562
		22.5°C			25°C	
wild-type (N2)	100	0	1213	100	0	389
asm-3(ok1744)	100	0	1194	100	0	484
daf-7(e1372)	38	62	993	0	100	430
asm-3(ok1744);daf-7(e1372)	40	60	1437	0	100	518
